# Extracellular ATP promotes endocrine resistance in ER+ breast cancer through upregulation of PYGL

**DOI:** 10.1038/s41419-026-08736-8

**Published:** 2026-04-13

**Authors:** Yu-Qing Yu, Xin-Yao Yu, Xiao-Fei Li, Yi-Fan Cheng, Yan-Ting Zhou, Fang Mei, Liang Weng, Xin-Xia Tian

**Affiliations:** Department of Pathology, School of Basic Medical Sciences, Peking University Third Hospital, Peking University Health Science Center, Beijing, China

**Keywords:** Breast cancer, Cancer metabolism, Prognostic markers, Cancer therapeutic resistance

## Abstract

Hormone receptor (HR)–positive breast cancer accounts for approximately 60% of all breast cancer cases, for which endocrine therapy represents the mainstay of treatment; however, the development of therapeutic resistance substantially limits its clinical efficacy. Extracellular adenosine 5′-triphosphate (ATP) has been implicated as a key mediator of metastasis and chemotherapy resistance in multiple malignancies, including breast cancer, yet its role in endocrine resistance remains poorly defined. Here, we demonstrate that extracellular ATP upregulates glycogen phosphorylase L (PYGL) expression in ER-positive breast cancer cells following endocrine treatment, thereby promoting endocrine resistance. Mechanistically, extracellular ATP activates the P2Y12–AhR signaling axis, leading to increased PYGL expression, enhanced glycolytic activity, and subsequent resistance to endocrine therapy. Moreover, elevated PYGL expression was strongly associated with reduced endocrine therapy sensitivity in breast cancer organoids and clinical tumor specimens. Collectively, these findings identify extracellular ATP–driven PYGL activation as a critical mechanism underlying endocrine resistance and suggest that targeting this pathway may represent a promising strategy to improve endocrine therapy efficacy in breast cancer patients.

## Introduction

Globally, breast cancer remains the most commonly diagnosed malignancy among women and the second leading cause of cancer-related mortality worldwide [1]. Hormone receptor (HR)–positive breast cancer accounts for approximately 60% of all diagnosed cases, for which endocrine therapy represents the cornerstone of systemic treatment [2, 3]. Among endocrine agents, tamoxifen—a selective estrogen receptor modulator (SERM) approved by the U.S. Food and Drug Administration in 1978—has been extensively used across all stages of HR-positive breast cancer, including adjuvant, neoadjuvant, and metastatic settings [4]. Fulvestrant, a selective estrogen receptor degrader (SERD), is commonly administered as first-line or subsequent endocrine therapy in postmenopausal patients with advanced disease following failure of tamoxifen or aromatase inhibitors [5, 6]. Although endocrine therapy substantially reduces recurrence rates and breast cancer–related mortality, its long-term efficacy is severely compromised by the development of intrinsic or acquired resistance.

Multiple molecular mechanisms underlying endocrine resistance have been proposed, including ESR1 mutations, aberrant activation of the MAPK and PI3K/AKT signaling pathways, and alterations in MYC-associated transcriptional programs [7]. Nevertheless, despite these advances, approximately 60% of endocrine resistance mechanisms remain poorly understood, underscoring the need to identify additional regulatory pathways that contribute to therapeutic failure.

Emerging evidence indicates that the tumor microenvironment (TME) plays a critical role in shaping endocrine therapy responses. Our group previously reported that extracellular adenosine 5′-triphosphate (ATP, eATP) functions as a potent pro-invasive and pro-chemoresistance factor within the TME [8]. Under physiological conditions, extracellular ATP concentrations (10–100 nM) are markedly lower than intracellular ATP levels (3–5 mM) [9]. However, in the tumor microenvironment, hypoxia, mechanical stress, and cell damage induce massive ATP release from tumor and stromal cells, resulting in extracellular ATP concentrations that can reach several hundred micromolar [10]. Elevated eATP levels have been shown to promote invasion and metastasis in prostate and breast cancers [11–13], and to modulate breast cancer progression by influencing angiogenesis and fibroblast activation within the TME [14, 15]. Moreover, extracellular ATP is capable of activating the PI3K/AKT and MAPK signaling pathways [16, 17], both of which are well-established contributors to endocrine resistance. These observations prompted us to investigate whether eATP directly participates in the regulation of endocrine resistance in breast cancer.

Tumor microenvironment remodeling frequently cooperates with metabolic reprogramming to support tumor progression and influence therapeutic responses. Glycogen phosphorylase L (PYGL), a member of the glycogen phosphorylase (GP) family, is a rate-limiting enzyme in glycogen catabolism that catalyzes the conversion of glycogen to glucose-1-phosphate, thereby fueling glycolysis. The GP family comprises three isoforms: PYGM (muscle), PYGL (liver), and PYGB (brain) [18, 19]. Increasing evidence suggests that PYGL is aberrantly upregulated in multiple malignancies, including clear cell renal cell carcinoma, papillary renal cell carcinoma, seminoma, glioblastoma, and other brain tumors, where it promotes tumor cell proliferation, invasion, metastasis, and resistance to chemotherapy [20–23]. In pancreatic cancer, PYGL-driven metabolic reprogramming facilitates epithelial–mesenchymal transition and metastatic dissemination [24]. Similarly, PYGL enhances tumor progression, metastasis, and chemoresistance in head and neck squamous cell carcinoma through modulation of the GSH/ROS/p53 axis [25]. Despite these findings, whether PYGL-mediated metabolic reprogramming contributes to endocrine resistance in breast cancer—and how such regulation is influenced by extracellular cues within the tumor microenvironment—remains largely unexplored.

In the present study, we identify extracellular ATP as a critical driver of endocrine resistance in ER-positive breast cancer through comprehensive functional and molecular analyses. Exposure to eATP consistently reduced sensitivity to endocrine therapies across multiple in vitro and in vivo assays. Transcriptomic profiling further revealed PYGL as a pivotal mediator of ATP-induced endocrine resistance, with its upregulation validated across multiple ER-positive breast cancer models using complementary experimental approaches. Based on these findings, we propose that PYGL functions as a key downstream effector of eATP signaling and contributes to metabolic reprogramming that supports endocrine resistance. We further delineate the molecular mechanisms underlying this process, thereby uncovering a previously unrecognized link between extracellular ATP signaling, metabolic adaptation, and endocrine therapy resistance in breast cancer.

## Materials and Methods

### Cell lines and cell culture

The human breast cancer cell lines MCF7 and ZR-75-1 were purchased from the Cell Resource Center, Institute of Basic Medical Sciences, Chinese Academy of Medical Sciences, and cultured in HDMEM or RPMI-1640 medium supplemented with 10% fetal bovine serum (FBS). The MCF7/TamR cell model was established by culturing MCF7 cells in medium containing 1 μM tamoxifen citrate (Sigma-Aldrich, T9262-1G) for 6 months, as previously described. All cells were maintained at 37 °C with 5% CO_2_. To generate shPYGL cells, two different shRNA hairpins targeting human PYGL were cloned into LKO.1 and used to knock down PYGL constitutively in various breast cancer cell lines. All cell lines were authenticated by short tandem repeat (STR) profiling and routinely tested for mycoplasma contamination.

### Cell proliferation and viability assays

Cell proliferation was assessed by plating 2000 cells per well in 96-well plates using either HDMEM or RPMI-1640 medium supplemented with 10% FBS, penicillin/streptomycin, and L-glutamine. After 24 hours, one plate was harvested as a baseline (day 0) before treatment, while the remaining plates were subjected to different treatments, including vehicle (dimethyl sulfoxide), tamoxifen, and fulvestrant. For MCF7 and ZR-75-1 cells, plates were harvested on days 3, 5, and 7 post-seeding. Cell proliferation was measured using the Cell Counting Kit-8 (Yeasen).

The EdU (5-ethynyl-2’-deoxyuridine) incorporation assay was performed according to the manufacturer’s instructions (Beyotime Biotechnology, Shanghai, China). Cells were cultured in 24-well plates, and 50 μM EdU was added to each well for a 2 h incubation. Cells were then fixed with 4% paraformaldehyde and stained using Apollo and Hoechst 33342 dyes. The rate of EdU incorporation was quantified by calculating the proportion of EdU-positive cells relative to the total number of Hoechst-positive cells. Each experiment was performed in triplicate.

For cell cycle analysis, cells were collected, washed twice with ice-cold phosphate-buffered saline (PBS), and fixed with 70% ethanol at 4 °C for 24 h. After staining with Propidium Iodide (PI), samples were analyzed by flow cytometry.

### RNA-seq and data analysis

MCF7 cells were treated with tamoxifen, with or without 100 μM ATP for 4 days. Total RNA was extracted using Trizol (Invitrogen), and RNA-seq was performed by Beijing Genomics Institute (BGI). Gene Ontology (GO) and KEGG pathway analysis were conducted, and ATP-regulated metabolic genes in MCF7 cells were visualized in a heatmap.

### Breast cancer molecular subtype and survival analysis

Kaplan–Meier survival analysis was conducted using an online database (http://gepia2.cancer-pku.cn/), with data analyzed using a 35% cutoff in each case. Survival curves were plotted for 1071 patients based on PYGL protein expression. Detailed treatment sequence information was not uniformly available.

### TF binding site prediction

The nucleotide sequence of the PYGL promoter region (chr14:50,944,483–50,942,483) was extracted. TF binding site predictions were made using the JASPAR, PROMO, and Ominer databases. Candidate TFs were selected based on higher expression levels in ATP-stimulated MCF7 cells treated with tamoxifen (from RNA-seq data) and their relevance to breast cancer.

### Luciferase reporter assay

HEK293T cells were transiently transfected with the PYGL promoter region luciferase reporter (Luc), pcDNA3.1-FLAG-AhR, pcDNA3.1-FLAG-ELF3, pcDNA3.1-FLAG-HNF4G, or pcDNA3.1-FLAG, along with pRL-TK (Renilla luciferase, Promega) as an internal control. After 48 hours, luciferase activity was measured using a luminometer (Thermo Fisher Scientific), and firefly luciferase activity was normalized to Renilla luciferase activity. Experiments were conducted in triplicate.

### Patient-derived BC organoid model

For organoid culture, fresh tumor tissues were cut into 1–2 mm-diameter pieces and washed with cold PBS. Mammary tumors were minced and digested in DMEM/F12 containing collagenase type IV (300 U/ml), 0.2% trypsin, 5% FBS, and insulin (10 μg/ml) for 60 minutes at 37 °C with rotation. Tumor organoid suspensions were filtered through a 100-μm cell strainer and washed with serum-free DMEM/F12. Cells were resuspended in 50 μl of 70% Growth Factor Reduced (GFR) Matrigel Matrix (Corning) and plated in 24-well culture plates. After incubating for 10–15 minutes at 37 °C to solidify the Matrigel, the cells were cultured in Breast Cancer Organoid Kit (bioGenous, Soochow, China). Tumor samples were collected from breast cancer patients at Peking University Third Hospital. The use of postoperative tumor specimens and associated clinical data was approved by the Peking University Biomedical Ethics Committee (PUIRB-YS2025025), with a waiver of informed consent for this retrospective study involving de-identified samples.

### In vivo assay

Female Balb/c-nu mice, aged 5 weeks, were obtained from and bred in specific pathogen-free conditions at the Center of Experimental Animals (Peking University, Beijing, China). Sample sizes were based on prior experience and published studies, without formal power calculations, and were sufficient to detect reproducible biological effects. All procedures were approved by the Institutional Animal Care and Use Committee of Peking University (no. BCAA0306). ZR-75-1 cells (5 × 10⁶) were suspended in 50 μl of serum-free RMPI 1640 and injected subcutaneously. Tumor volumes were measured every three days. Animals were randomly allocated to experimental groups before treatment. Tamoxifen (20 mg/kg) was administered intraperitoneally every two days. After 55 days, all animals were sacrificed, and primary tumors were fixed in paraformaldehyde for hematoxylin and eosin (HE) and immunohistochemical (IHC) staining. IHC staining scores for PYGL, Ki67, and cleaved-caspase-3 were assessed. Blinding was not performed during the animal experiments.

### Statistical analysis

Experiments were conducted in triplicate. No samples or animals were excluded from the analyses. Data were analyzed using Prism 8 (GraphPad Software). Comparisons between two groups were conducted using two-tailed Student’s *t* tests, while differences among multiple groups were assessed by one-way analysis of variance (ANOVA). Data are presented as mean ± standard deviation (SD). Statistical significance was considered for *p*-values < 0.05. Data were assessed for normality and homogeneity of variance, and variability within each group was estimated. All statistical analyses were deemed appropriate for the respective datasets.

## Results

### Extracellular ATP promotes endocrine resistance

In the tumor microenvironment, extracellular ATP (eATP) concentrations range from several nM to a few hundred μM [10]. Our previous studies demonstrated that 100 μM ATP maximally enhances the invasiveness and migratory potential of human breast cancer cells [12–14]. We also found that 100 μM ATP was most effective in promoting tamoxifen resistance in MCF7 cells (Fig. S1A), and thus used this concentration for subsequent treatments in the current study. Although in vitro cultured breast cancer cells release eATP following tamoxifen-induced cell death, even without ATP supplementation, we measured eATP concentrations in the culture medium and found them to be around 25 nM (Fig. S1B), significantly lower than 100 μM. This suggests that the minor release of eATP from breast cancer cells under in vitro conditions can be disregarded. Since ATP is rapidly hydrolyzed to adenosine, we employed ATPγS (a non-hydrolyzable ATP analog) and adenosine to further investigate the role of ATP in endocrine resistance. We found that ATP and ATPγS, rather than adenosine, promoted endocrine resistance (Fig. S1C), confirming that eATP plays a critical role in resistance.

**Fig. 1:**
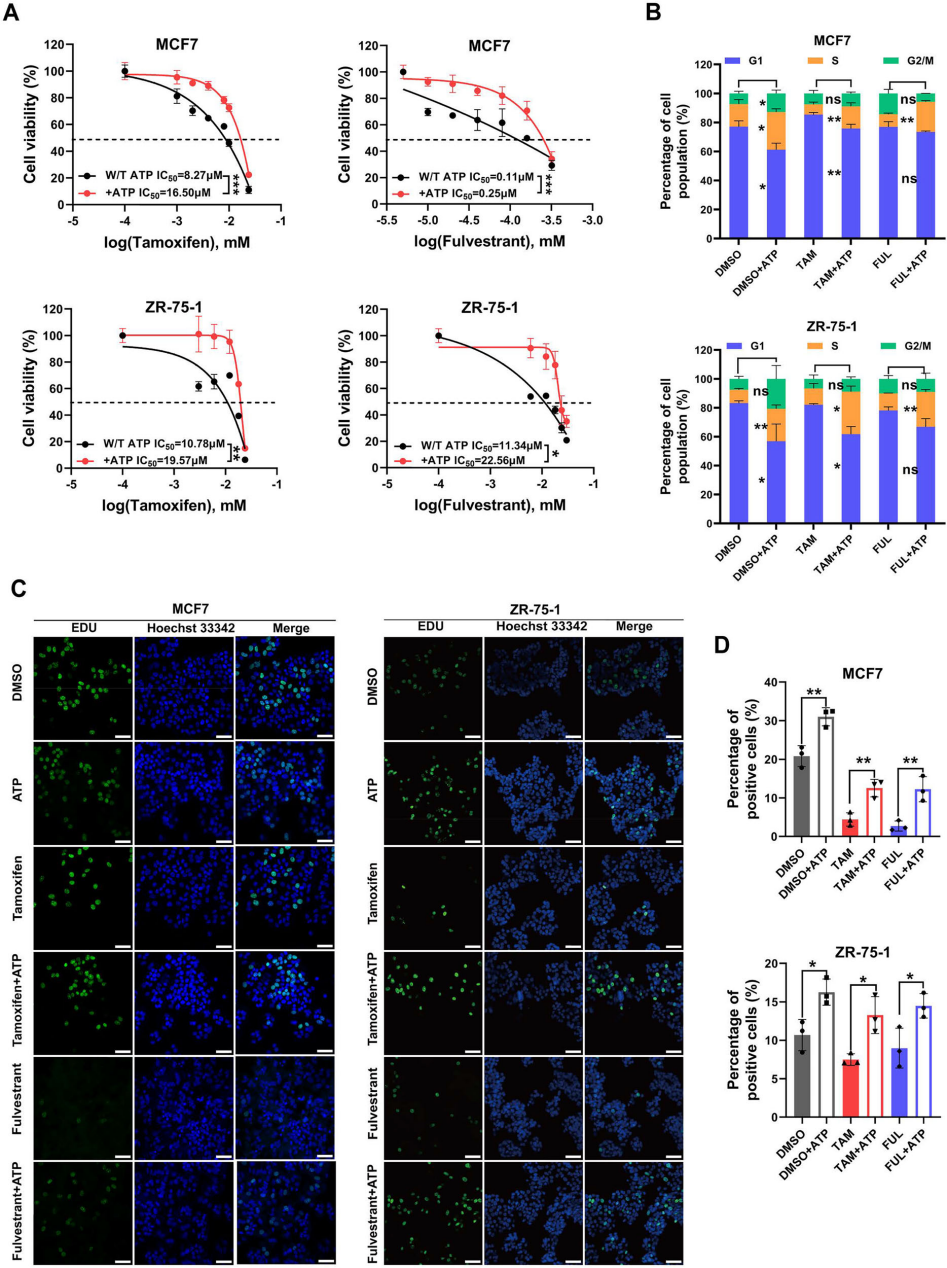
Extracellular ATP promotes endocrine resistance. **A** Dose-response curves for increasing doses of tamoxifen or fulvestrant in MCF7 and ZR-75-1 cells treated for 6 days with or without 100 μM ATP. Vehicle-treated control groups were set to 100% for each curve (*n* = 3). **B** Treatment with endocrine drugs plus ATP altered the distribution of MCF7 and ZR-75-1 cells across G1, S and G2/M phases, compared to endocrine drug alone treatment. Cells were treated for 6 days and analyzed by flow cytometry after PI staining (*n* = 3). **C** Representative images of EdU staining in MCF7 and ZR-75-1 cells following 6 days of treatment with endocrine drugs plus ATP or endocrine drugs alone. Scale bars, 50 μm. **D** Statistical analysis of EdU staining results (*n* = 3). Data represent at least three independent experiments. Statistical significance was assessed by a two-tailed unpaired t-test, one-way, and two-way ANOVA. Error bars represent SD; *p < 0.05; **p < 0.01; ***p < 0.001; ns not significant.

To further assess the impact of eATP on endocrine resistance, two ER+ breast cancer cell lines (MCF7 and ZR-75-1) were treated with tamoxifen plus ATP or fulvestrant plus ATP for 6 days. The CCK-8 assay revealed that ATP significantly enhanced endocrine resistance in both MCF7 and ZR-75-1 cells (Fig. 1A). Endocrine therapies are known to modulate the G1/S transition checkpoint by suppressing estrogen-mediated signaling [26]. Consistent with this, FACS analysis showed an increase in the number of cells in S phase and a decrease in G1 phase cells in MCF7 and ZR-75-1 cells treated with endocrine drugs plus ATP, compared with control cells treated with endocrine drugs alone (Fig. 1B). Additionally, the EdU incorporation assay demonstrated significantly enhanced proliferation of MCF7 and ZR-75-1 cells following treatment with endocrine drugs plus ATP (Fig. 1C, D). These findings indicate that ATP promotes endocrine resistance in ER+ breast cancer cells in vitro.

### PYGL is involved in ATP-driven endocrine resistance in ER+ breast cancer cells

**Fig. 2:**
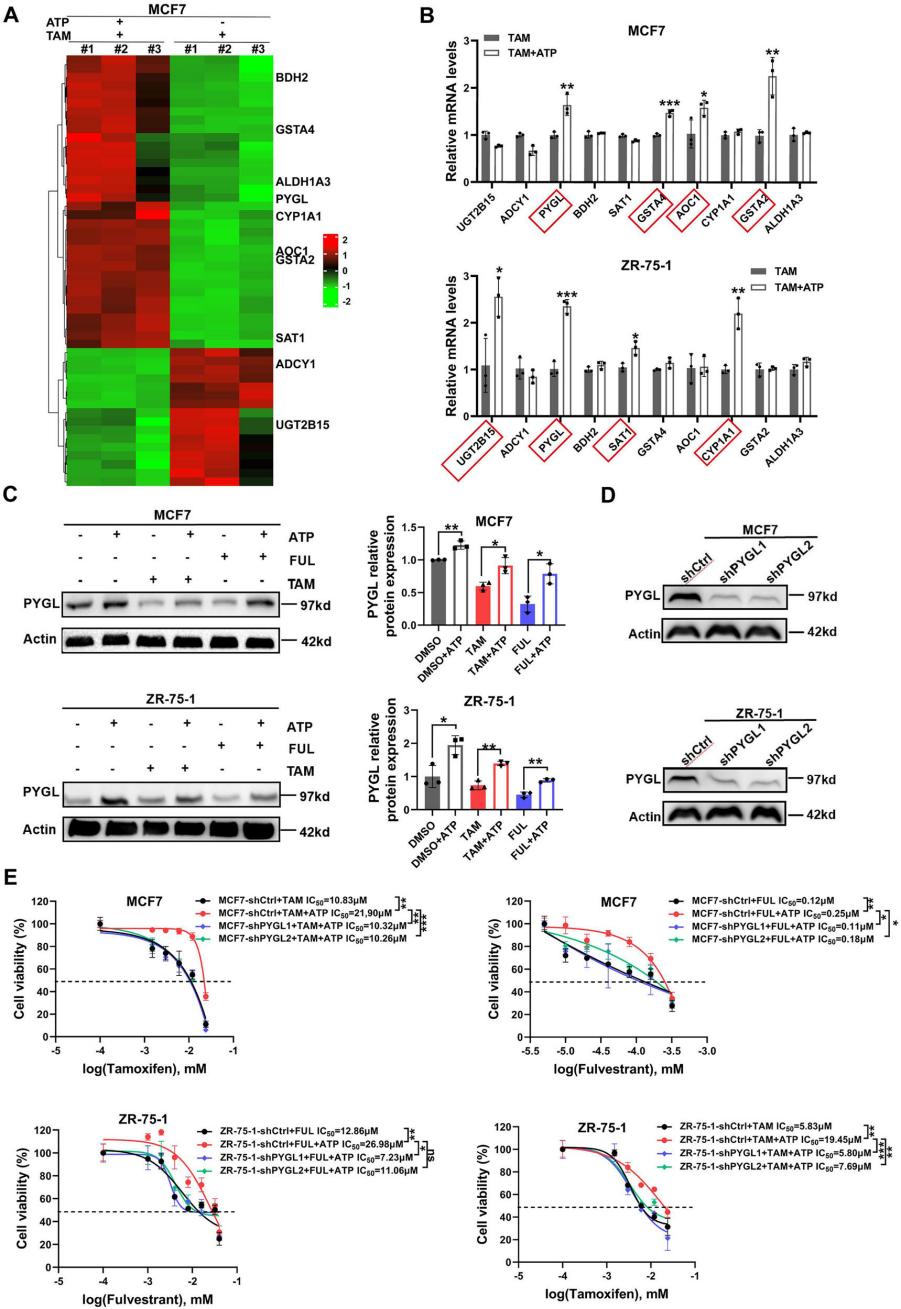
ATP up-regulates PYGL expression in ER+ breast cancer cells. MCF7 breast cancer cells were treated with or without 100 μM ATP and 5 μM tamoxifen for 4 days. Total RNA was extracted and RNA sequencing was performed to investigate the transcriptional profile in response to drug treatment. **A** Heatmaps depicting gene expression from RNA sequencing of MCF7 cells. Red and green colors represent gene expression levels above and below the mean, respectively. **B** mRNA levels of genes in metabolic pathways following ATP treatment were assessed by RT-qPCR in MCF7 and ZR-75-1 cells (*n* = 3). **C** PYGL protein expression was examined by Western blot after 6 days of treatment with DMSO, tamoxifen, or fulvestrant, with or without ATP (*n* = 3). **D** PYGL-knockdown cells were established in MCF7 and ZR-75-1 cells. **E** Growth curves of MCF7-shCtrl, MCF7-shPYGL, ZR-75-1-shCtrl, and ZR-75-1-shPYGL cells were measured by CCK-8 assay in monolayer culture (*n* = 3). Data represent at least three independent experiments. Statistical significance was assessed by a two-tailed unpaired t-test, one-way, and two-way ANOVA. Error bars represent SD; *p < 0.05; **p < 0.01; ***p < 0.001; ns not significant.

To investigate the molecular mechanisms underlying ATP-mediated resistance to tamoxifen in ER+ breast cancer (BC) cells, we performed RNA sequencing to identify differentially expressed genes between tamoxifen plus ATP and tamoxifen-only treatments (Table S1). KEGG enrichment analysis revealed that metabolic pathways were most significantly enriched (Fig. S2A). Among the differentially expressed genes, PYGL exhibited the largest change (2.3-fold increase) and was further validated in MCF7 and ZR-75-1 cells by RT-qPCR (Fig. 2A, B). Western blotting confirmed the upregulation of PYGL in cells treated with tamoxifen plus ATP or fulvestrant plus ATP compared to endocrine therapy alone (Fig. 2C). Moreover, Kaplan–Meier analyses of overall survival (OS) and disease-free survival (DFS) in BC patients from the GEPIA database (<span Type="Underline" Name="Emphasis" Class="Underline">GEPIA (Gene Expression Profiling Interactive Analysis)) revealed that high PYGL expression correlated with poorer OS and DFS in both BC and HR + BC patients (Fig. S2B). These findings led us to focus on PYGL for further investigation.

To assess the impact of PYGL on endocrine resistance, we treated control and PYGL knockdown cells with increasing doses of tamoxifen (Fig. 2D). PYGL knockdown increased sensitivity to both tamoxifen and fulvestrant in comparison to control cells (Fig. 2E). EdU staining assays (Fig. S3A) and cell cycle analysis (Fig. S3B) demonstrated that PYGL knockdown mitigated ATP-induced resistance in ER + BC cells. These results suggest that PYGL plays a pivotal role in ATP-driven resistance in ER + BC cells.

To further validate the role of PYGL in endocrine resistance, tamoxifen-resistant MCF7 cells (designated MCF7/TamR) were generated by treating cells with tamoxifen plus 100 μM ATP. Notably, MCF7/TamR cells exhibited no resistance to fulvestrant (Fig. S4A). Elevated PYGL mRNA levels were observed in MCF7/TamR cells compared to MCF7 cells (Fig. S4B). Knockdown of PYGL (Fig. S4C) significantly reduced resistance to tamoxifen, but not to fulvestrant, as evidenced by decreased cell viability (Fig. S4D), a reduced proportion of cells in the S phase (Fig. S4E), and fewer EdU-positive cells (Fig. S4F). These findings confirm that PYGL is a critical regulator of tamoxifen resistance in ER + BC cells.

### The eATP-driven endocrine resistance is via glycolysis mediated by PYGL

As a member of the glycogen phosphorylase (GP) family, PYGL is a key enzyme in glycolysis through the glycogen degradation pathway, catalyzing the release of glucose-1-phosphate from glycogen. Previous studies have linked increased glycolysis to chemoresistance in various human cancers [27]. We then investigated whether PYGL-regulated glycolysis contributes to ATP-driven endocrine resistance in ER+ breast cancer.

**Fig. 3:**
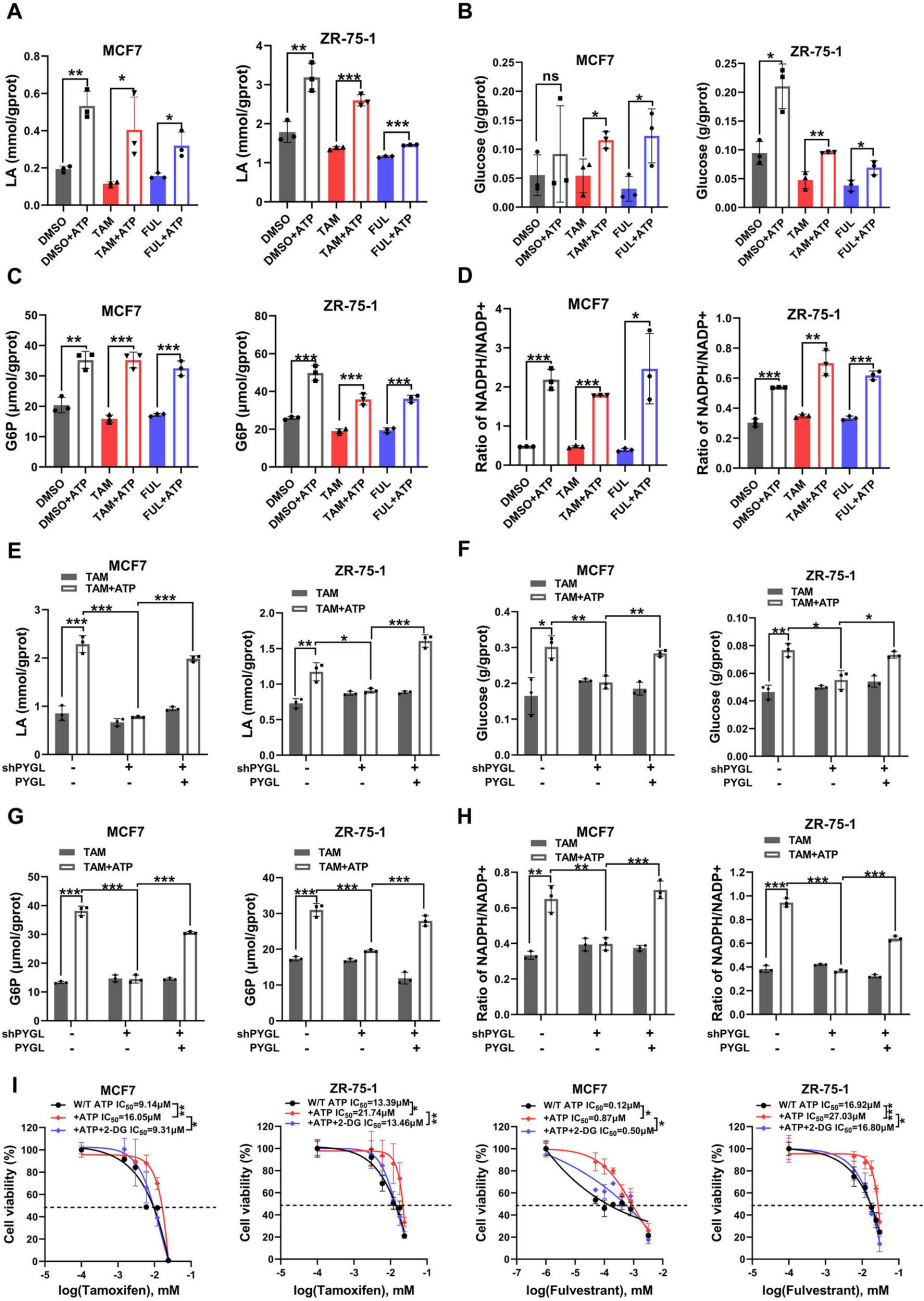
PYGL is involved in ATP-driven glycolysis. **A**–**D** The production of lactate, glucose, G6P and the NADPH/NADP+ ratio in MCF7 and ZR-75-1 cells was significantly increased after treatment with endocrine drugs plus ATP, compared to treatment with endocrine drugs alone (*n* = 3). **E**–**H** PYGL knockdown attenuated the ATP-driven increases in lactate, glucose, G6P and the NADPH/NADP+ ratio in MCF7 and ZR-75-1 cells, while PYGL overexpression reversed this effect (*n* = 3). **I** 2-DG, a glycolytic pathway inhibitor, reversed ATP-induced endocrine resistance in MCF7 and ZR-75-1 cells (*n* = 3). Data are representative of at least three independent experiments. Statistical significance was assessed using a two-tailed unpaired t-test, one-way and two-way ANOVA. Error bars represent SD; *p < 0.05; **p < 0.01; ***p < 0.001; ns not significant.

We observed elevated levels of lactate (Fig. 3A), glucose (Fig. 3B), glucose-6-phosphate (G6P) (Fig. 3C) and the NADPH/NADP+ ratio (Fig. 3D) in MCF7 and ZR-75-1 cells treated with endocrine therapy plus ATP, compared to those treated with endocrine therapy alone, indicating that ATP upregulates glycolysis. Interestingly, specific knockdown of PYGL significantly attenuated the ATP-induced increases in lactate, glucose, G6P, and NADPH/NADP+ in both MCF7 and ZR-75-1 cells, an effect that was reversed by PYGL overexpression (Figs. 3E–H, S5B, C). These results suggest that PYGL plays a functional role in the ATP-driven enhancement of glycolysis in endocrine-resistant ER+ breast cancer cells.

We further evaluated the impact of ATP and PYGL on glycogen content in MCF7 cells using the fluorescent glucose analog 2-NBDG. ATP treatment significantly decreased glycogen levels, while the PYGL inhibitor CP91149 increased glycogen content (Fig. S5A). Additionally, inhibition of glycolysis by 2-deoxy-D-glucose (2-DG) markedly reduced the ability of ATP to promote endocrine resistance in both MCF7 and ZR-75-1 cells (Fig. 3I). Similar results were observed in MCF7/TamR cells (Fig. S5D, E). Collectively, these data indicate that PYGL-mediated glycolysis contributes to ATP-driven endocrine resistance in ER+ breast cancer.

### ATP modulates PYGL signaling via the P2Y12-AhR axis

**Fig. 4:**
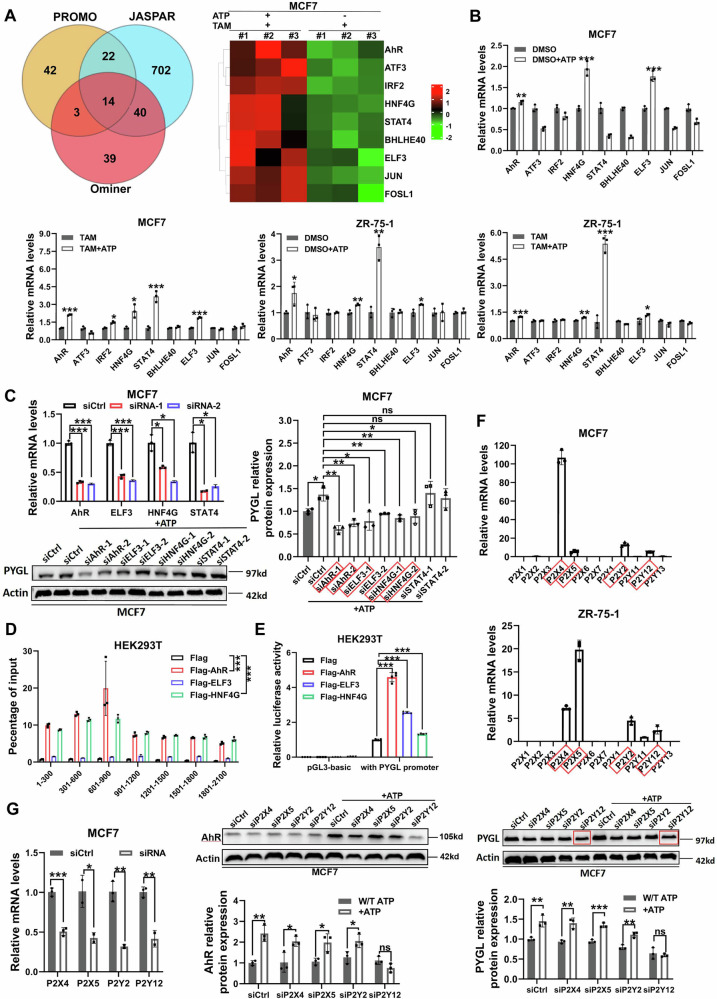
ATP modulates PYGL signaling via P2Y12-AhR axis. **A** Left: Venn diagrams showing the overlap of potential upstream transcription factors (TFs) predicted by the PROMO, JASPAR and Ominer databases. Right: Heatmaps of the predicted upstream TFs identified in MCF7 RNA-sequencing analysis. MCF7 cells were treated with tamoxifen plus ATP or tamoxifen alone. **B** The mRNA expression levels of the predicted upstream TFs in MCF7 and ZR-75-1 cells following the indicated treatments were analyzed by RT-qPCR (*n* = 3). **C** Western blotting confirmed that ATP-driven upregulation of PYGL was attenuated by siRNAs targeting specific TFs (*n* = 3). **D** Flag-AhR, Flag-ELF3, or Flag-HNF4G were transfected into HEK293T cells. The different PYGL promoter regions pulled down with Flag antibody in HEK293T cells were analyzed by RT-qPCR (*n* = 3). **E** Luciferase reporter assays were performed following transient co-transfection of HEK293T cells with the indicated constructs. The PYGL promoter was cloned into a luciferase reporter vector and co-transfected with an empty vector (pcDNA3.1) or vectors overexpressing the predicted upstream TFs (pcDNA3.1-AhR, pcDNA3.1-ELF3, pcDNA3.1-HNF4G) (*n* = 4). **F** The mRNA expression levels of ATP receptors in MCF7 and ZR-75-1 cells were analyzed by RT-qPCR (*n* = 3). **G** RT-qPCR analysis of ATP receptor knockdown efficiency in MCF7 cells. Western blotting showed that ATP-driven upregulation of AhR and PYGL was attenuated by siP2Y12 (*n* = 3). Data are representative of at least three independent experiments. Statistical significance was determined using two-tailed unpaired t-test and one-way ANOVA. Error bars represent SD; *p < 0.05; **p < 0.01; ***p < 0.001; ns not significant.

In order to gain insight into the molecular mechanism of ATP modulating PYGL, we first analyzed the upstream transcription factors of PYGL using the following three online tools: https://alggen.lsi.upc.es/cgi-bin/promo_v3/promo/promoinit.cgi?dirDB=TF_8.3; http://signalingpathways.org/ominer/query.jsf; https://jaspar.elixir.no/. Fourteen TFs were predicted as candidates, and RNA-seq data revealed that the mRNAs of nine TFs were upregulated in MCF7 cells treated with tamoxifen plus ATP, compared to tamoxifen alone (Fig. 4A). RT-qPCR confirmed that AhR, ELF3, HNF4G, and STAT4 were upregulated following ATP treatment (Fig. 4B). Western blotting further showed that knockdown of AhR, ELF3, or HNF4G downregulated PYGL expression (Fig. 4C).

To identify which TF promote PYGL expression, we constructed firefly luciferase reporter plasmids containing the PYGL promoter and performed chromatin immunoprecipitation (ChIP)–qPCR. As shown in Fig. 4D, AhR and HNF4G were found to bind to the PYGL promoter. Moreover, the relative luciferase activity was highest in HEK293T cells co-transfected with the PYGL promoter and AhR (Fig. 4E), suggesting that AhR regulates PYGL transcription. Western blotting also confirmed increased AhR expression following tamoxifen plus ATP or fulvestrant plus ATP treatment compared with endocrine therapy alone in ER + BC cells (Fig. S6A). Additionally, ChIP–qPCR analysis confirmed that ATP treatment augmented AhR occupancy at the PYGL locus, validating the molecular mechanism of the ATP–AhR–PYGL axis (Fig. S6B).

To test the functional relevance of AhR in ATP-driven resistance, we treated BC cells with the AhR inhibitor CH-223191. CCK-8 assays showed that CH-223191 increased sensitivity to endocrine therapy in both MCF7 and ZR-75-1 cells (Fig. S6C). EdU incorporation assays further demonstrated that cellular proliferation was significantly inhibited in these cells after treatment with endocrine drugs plus ATP and CH-223191 (Fig. S6D). Moreover, CH-223191 restored tamoxifen sensitivity in MCF7/TamR cells (Fig. S7A, B). These findings suggest that ATP-induced upregulation of AhR enhances PYGL expression, thereby promoting resistance.

Our previous studies have shown that P2 receptors, in response to eATP, activate downstream signaling pathways such as MAPK, promoting invasion and metastasis in breast cancer cells [12, 16]. We next investigated which P2 receptor is involved in the ATP-AhR-PYGL signaling pathway. RT-qPCR analysis of P2 receptor expression in MCF7 and ZR-75-1 cells revealed that P2X4, P2X5, P2Y2 and P2Y12 were significantly more highly expressed than other P2 receptors (Fig. 4F). Knockdown of P2X4, P2X5, P2Y2, or P2Y12 in MCF7 cells showed that ATP-driven upregulation of AhR and PYGL was significantly attenuated by P2Y12 knockdown (Fig. 4G). Elevated P2Y12 expression in MCF7/TamR cells further suggested activation of the P2Y12-AhR-PYGL signaling pathway, potentially contributing to tamoxifen resistance (Fig. S7C). Collectively, these data confirm that ATP promotes PYGL expression in BC cells via the P2Y12-AhR axis.

### The sensitivity of ER+ breast cancer (BC) organoids to endocrine therapy is correlated with the level of PYGL expression

**Fig. 5:**
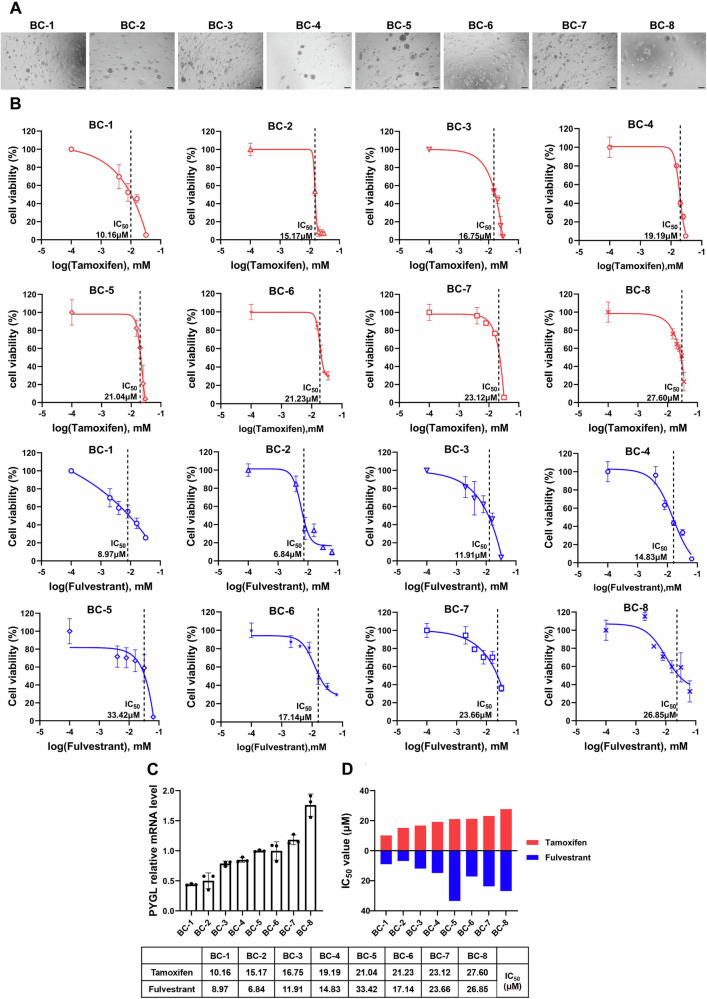
The endocrine therapy sensitivity of ER + BC organoids is related to the level of PYGL expression. **A** Representative brightfield images of 8 breast cancer organoids. **B** IC_50_ curves for the treatment of 8 breast cancer organoids with tamoxifen and fulvestrant (*n* = 3). **C** RT-qPCR analysis of PYGL expression levels in 8 breast cancer organoids (*n* = 3). **D** Upper panel: Two-way bar chart showing similar sensitivity to tamoxifen and fulvestrant in the 8 breast cancer organoids, as indicated by IC_50_ values; lower panel: IC_50_ values for the 8 breast cancer organoids as shown. Data are representative of at least three independent experiments. Error bars represent SD.

Given the frequent development of resistance to tamoxifen and fulvestrant [7], we examined the clinical relevance of our findings in the context of endocrine therapy. Fresh breast cancer biopsy specimens were collected, and patient-derived breast cancer (BC) organoids were successfully established (Fig. 5A and Table S2). Cell viability assays were performed on 8 BC organoid lines treated with tamoxifen or fulvestrant (Fig. 5B), and PYGL expression levels were quantified by RT-qPCR (Fig. 5C). Organoids with lower PYGL expression displayed increased sensitivity to endocrine therapy, as indicated by lower IC₅₀ values (Fig. 5D).

### PYGL promotes xenograft tumor tamoxifen resistance and glycolysis in vivo

**Fig. 6:**
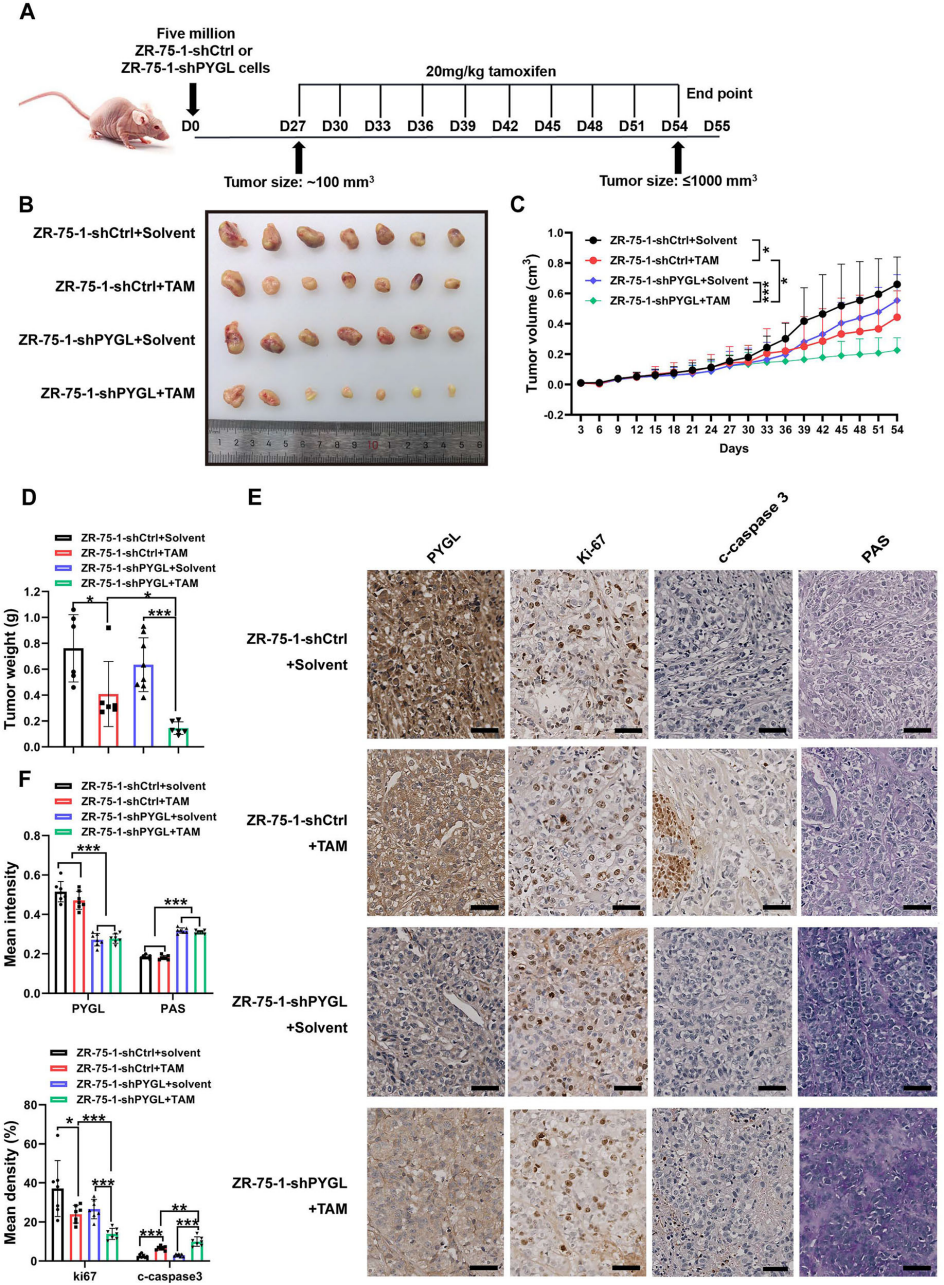
PYGL promotes xenograft tumor tamoxifen resistance and glycolysis in vivo. Stably transfected ZR-75-1 cells (shCtrl, shPYGL) were subcutaneously inoculated into 5-week-old female Balb/c mice (*n* = 7) and treated with tamoxifen once the average tumor volume reached 100 mm³. **A** Schematic diagram of the murine xenograft tumor model. **B** Bright-field images of excised subcutaneous tumors (*n* = 7). **C** Tumor volume was measured and quantified every 3 days. **D** Weights of excised primary tumors. **E** Tumor tissues were subjected to immunohistochemical (IHC) staining. Scale bar, 50 µm. **F** Quantification of IHC staining was performed using ImageJ software. Statistical analysis was conducted using one-way and two-way ANOVA. Error bars represent SD; *p < 0.05; **p < 0.01; ***p < 0.001.

To investigate the role of PYGL in tumor sensitivity to endocrine therapy in vivo, 5 × 10⁶ ZR-75-1 cells stably transfected with PYGL shRNA (shPYGL group, *n* = 14) or control shRNA (shCtrl group, *n* = 14) were subcutaneously injected into Balb/c mice. On day 27 post-inoculation, when tumor volumes reached approximately 100 mm³, the mice were treated with tamoxifen or vehicle (20 mg/kg, intraperitoneal injection). Tumor growth was monitored every three days for 8 weeks following cell injection (Fig. 6A).

Compared to the control group, tamoxifen-treated mice in the shPYGL group exhibited significantly reduced tumor progression, as indicated by smaller tumor volumes (Fig. 6B), slower tumor growth rates (Fig. 6C) and decreased tumor weights (Fig. 6D). Similar antitumor effects were observed in the shP2Y12 and shAhR groups (Fig. S8). Moreover, tamoxifen treatment in the shPYGL group correlated with reduced Ki-67 expression (indicating decreased proliferation) and increased cleaved caspase-3 levels (indicating enhanced apoptosis), further validating the enhanced therapeutic response. Histological analysis also revealed significantly increased glycogen deposits in shPYGL tumors (Fig. 6E, F), directly supporting PYGL’s role in glycogenolytic regulation. Collectively, these results suggest that PYGL depletion enhances tumor sensitivity to tamoxifen.

### PYGL may serve as a predictor of endocrine therapy resistance

**Fig. 7:**
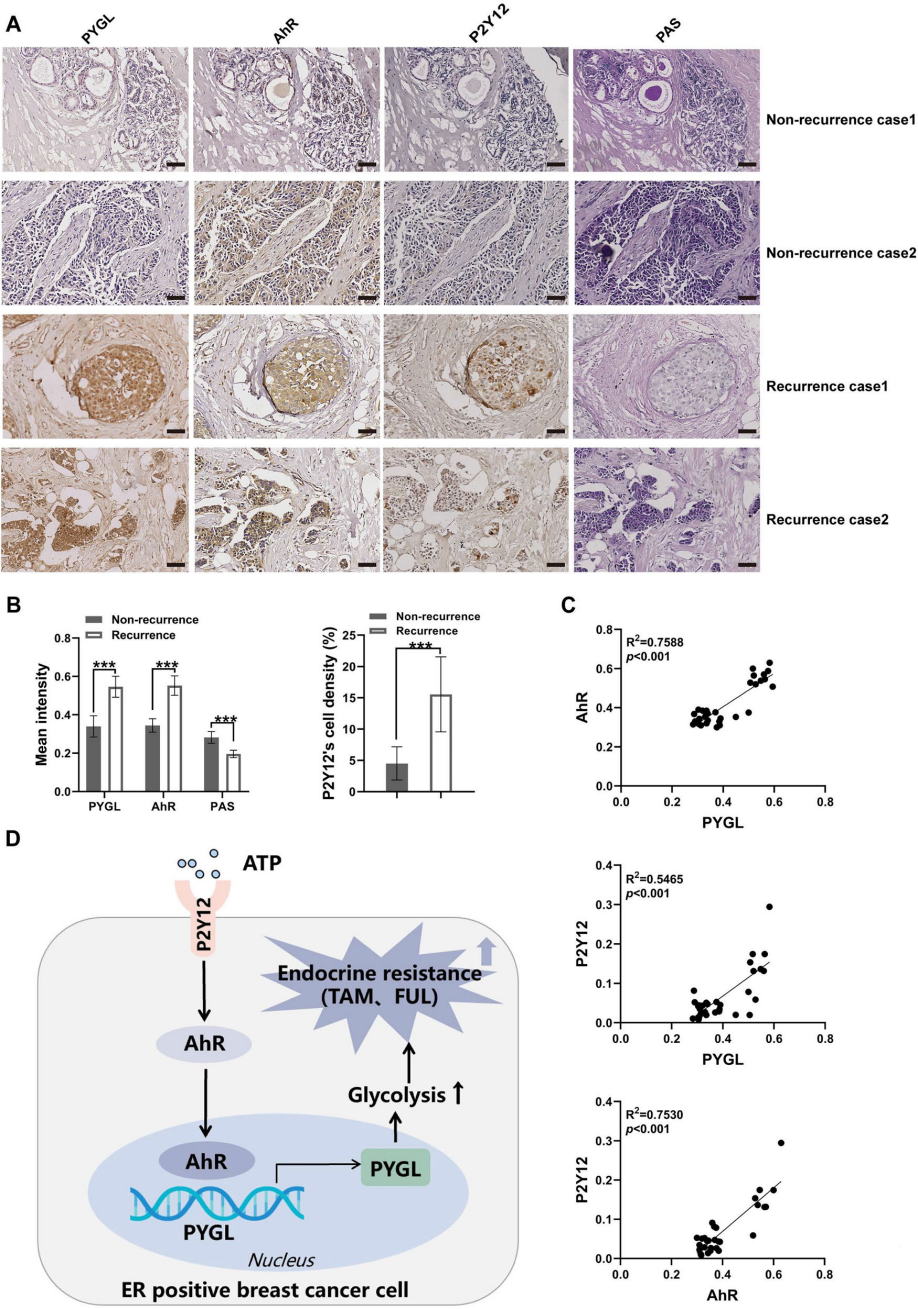
PYGL may serve as a predictor of endocrine therapy resistance. **A** Immunohistochemical (IHC) staining showed that breast cancer tissues from patients with recurrence exhibited upregulated expression of ATP-PYGL axis-related molecules and reduced glycogen content compared to tissues from recurrence-free patients over a 6-year follow-up. Scale bar, 50 µm. **B** IHC data were analyzed using ImageJ software. **C** Correlation analysis revealed positive pairwise correlations between expression levels of molecules P2Y12, AhR, and PYGL in breast cancer cases. **D** The proposed model for the ATP-PYGL signaling. Data are represented as means ± SD. *P*-value is determined by the t-test (two-sided) or the Log-rank test. ***p < 0.001.

To investigate the clinical relevance of PYGL in endocrine therapy resistance in breast cancer patients, we analyzed the expression of PYGL, AhR and P2Y12 proteins, as well as glycogen levels, in a cohort of 34 breast cancer specimens, including 10 cases with confirmed post-treatment recurrence or metastasis and 24 recurrence-free cases (Table S3). Immunohistochemical analysis revealed significantly elevated levels of PYGL, AhR, and P2Y12 in recurrence/metastasis specimens compared to non-recurrent cases. Notably, these samples also exhibited reduced glycogen content, consistent with PYGL’s known role in glycogenolysis (Fig. 7A, B). Furthermore, the expression of PYGL, AhR, and P2Y12 was positively correlated in the breast cancer samples (Fig. 7C). These findings validate our previous experimental observations on the role of the P2Y12–AhR–PYGL axis in therapeutic resistance mechanisms (Fig. 7D).

## Discussion

Although ATP has been extensively studied as an energy substrate, its role in cancer, particularly extracellular ATP (eATP), remains underexplored [28, 29]. While previous studies have demonstrated that eATP promotes cancer invasion and chemotherapy resistance [8], its involvement in endocrine resistance is not well understood. This study aimed to investigate the role of eATP in endocrine resistance.

Among various eATP concentrations, 100 μM ATP exhibited the most pronounced effect on promoting tamoxifen resistance. This effect may be attributed to the activation of distinct receptor subtypes by different ATP concentrations, which can either inhibit or promote cancer growth. Specifically, P2Y1 and P2Y2 receptors promote cancer growth, whereas P2X7 exerts inhibitory effects [30]. Consistent with our findings, 100 μM ATP significantly increased intracellular Ca²⁺ levels, thereby activating downstream signaling pathways [31]. Based on these results, 100 μM ATP was used in subsequent functional experiments and RNA-seq analyses.

RNA-seq and KEGG pathway analysis of tamoxifen-treated MCF7 cells revealed that ATP significantly upregulated the expression of several metabolism-related genes. Previous studies have shown that ATP suppresses gluconeogenesis and lipid accumulation in hepatocytes via P2Y receptors, supporting our findings [32]. We selected PYGL for further investigation for several reasons: (i) PYGL is a key enzyme in glycolysis, influencing tumor development both in vitro and in vivo by modulating glycolytic activity [20]. It also affects sensitivity to radiotherapy and chemotherapy, making it a potential therapeutic target [21, 25]. However, its role in endocrine therapy remains unclear. (ii) RT-qPCR and Western blot validation showed that PYGL expression was significantly upregulated in ER-positive breast cancer cell lines treated with 100 μM ATP and endocrine drugs. (iii) Survival analyses of public datasets showed that high PYGL expression was associated with poorer overall and disease-free survival in breast cancer patients. The biphasic survival pattern may reflect temporal changes in endocrine therapy, as PYGL modulates tamoxifen sensitivity but does not confer cross-resistance to fulvestrant in tamoxifen-resistant cells. However, definitive stratification is limited by incomplete treatment sequence information. These findings prompted further investigation into the relationship between eATP and PYGL in cancer cells, providing the first evidence that eATP regulates PYGL expression and contributes to endocrine resistance.

Even under aerobic conditions, tumor cells often shift from mitochondrial oxidative metabolism to glycolysis. As a key enzyme in glycogenolysis, PYGL plays a critical role in glycolytic reprogramming, a function validated in this study. Extracellular purines are known to influence metabolic reprogramming through various pathways [33], with glycolytic enzymes and metabolites, such as lactate and ATP, modulating cancer progression and therapeutic resistance by interfering with cell cycle regulation [27, 34–37]. Additionally, metabolic reprogramming occurs in a cell cycle phase-specific manner: the tricarboxylic acid (TCA) cycle predominates in the G1 phase, while glycolysis is the primary energy source in the S phase [38]. Our study shows that eATP-induced PYGL upregulation promotes glycolysis, cell cycle redistribution, and endocrine resistance in breast cancer cells.

Given that both mRNA and protein levels of PYGL were upregulated following ATP stimulation, we hypothesized that ATP activates a transcription factor that regulates PYGL expression. Through analysis of public databases and RNA-seq data, we identified potential transcription factors that bind to the PYGL promoter. ChIP-qPCR and luciferase assays revealed that the AhR transcription factor activates PYGL transcription. This is consistent with a study showing that PYGL is activated by AhR ligands in breast cancer [39, 40]. AhR, a promising therapeutic target in breast cancer [41], regulates various processes, including cell proliferation, metabolism, and stem cell characteristics [42–45]. Additionally, AhR has been implicated in mediating chemotherapy resistance through reactive oxygen species (ROS) [46], further supporting our results. Other factors, such as RIP3 [47] and HIF1α [48], also regulate PYGL expression. Given the heterogeneity of breast cancer, various intercellular and extracellular factors may influence PYGL expression across different conditions or tumor stages. In summary, we identify AhR as a key transcription factor regulating PYGL expression in breast cancer cells.

ATP released by cells activates P2 receptors through paracrine or autocrine signaling [49]. Although P2Y12 receptor expression is not predominant in most breast cancer cell lines, our study shows that ATP activates the AhR-PYGL signaling axis by stimulating P2Y12 receptors, which are highly expressed in MCF7/TamR cells. This finding aligns with previous evidence showing that ATP activates various signaling pathways by engaging specific receptors [30]. As a Gi-coupled GPCR, P2Y12 has been shown to influence tumor proliferation and metastasis [50, 51], and its inhibition enhances chemotherapy-induced cell death and apoptosis [52], consistent with our observations.

Our mouse model demonstrated that PYGL shRNA enhanced tamoxifen sensitivity and increased glycogen levels in implanted tumors. Similarly, PYGL knockdown inhibited tumor xenograft growth in mice by inducing senescence [20], while liver-specific PYGL knockout promoted glycogen accumulation in the liver [53]. These in vivo results support our findings. Moreover, organoid drug sensitivity assays and clinical cohort analyses confirmed that high PYGL expression is associated with reduced endocrine therapy sensitivity, further highlighting PYGL as a potential therapeutic target for overcoming endocrine resistance in breast cancer. Notably, small-molecule inhibitors of PYGL, such as CP91149 and CP320626, are currently used for diabetes treatment but have not yet been explored for breast cancer therapy [54, 55], underscoring the potential of PYGL-targeted therapies.

Despite these findings, our study has limitations. The genetic heterogeneity of the MCF7/TamR model and the relatively small size of the clinical cohorts restrict comprehensive mechanistic insights and generalizability. Future studies should validate these observations in larger cohorts of breast cancer patients undergoing endocrine therapy.

Overall, our study identifies the P2Y12–AhR–PYGL axis as a key mediator of extracellular ATP–induced endocrine resistance in ER-positive breast cancer through glycolytic reprogramming (Fig. 7D). The elevated expression of PYGL observed in endocrine-resistant patient tumors and breast cancer organoids was associated with reduced sensitivity to endocrine therapy, supporting its functional involvement in therapeutic failure. Mechanistically, PYGL acts as a downstream effector of P2Y12–AhR signaling, linking extracellular ATP sensing to metabolic adaptation under tamoxifen stress. Collectively, these findings highlight PYGL and its upstream regulatory pathway as clinically relevant therapeutic vulnerabilities and potential targets for overcoming endocrine resistance.

## Supplementary information

Supplementary figures; Supplementary tables; Supplementary methods

the original western blot

## Data Availability

All data and materials during the current study are available from the corresponding author upon reasonable request.
