# Supplementary material for: Extracellular ATP promotes endocrine resistance in ER+ breast cancer through upregulation of PYGL
Source: Cell Death Dis. 2026 Apr 13;17(1):476. doi: 10.1038/s41419-026-08736-8 (PMC13184142; doi:10.1038/s41419-026-08736-8)
Supplement: Supplementary file 1 — Supplementary figures; Supplementary tables; Supplementary methods [file 41419_2026_8736_MOESM1_ESM.pdf]

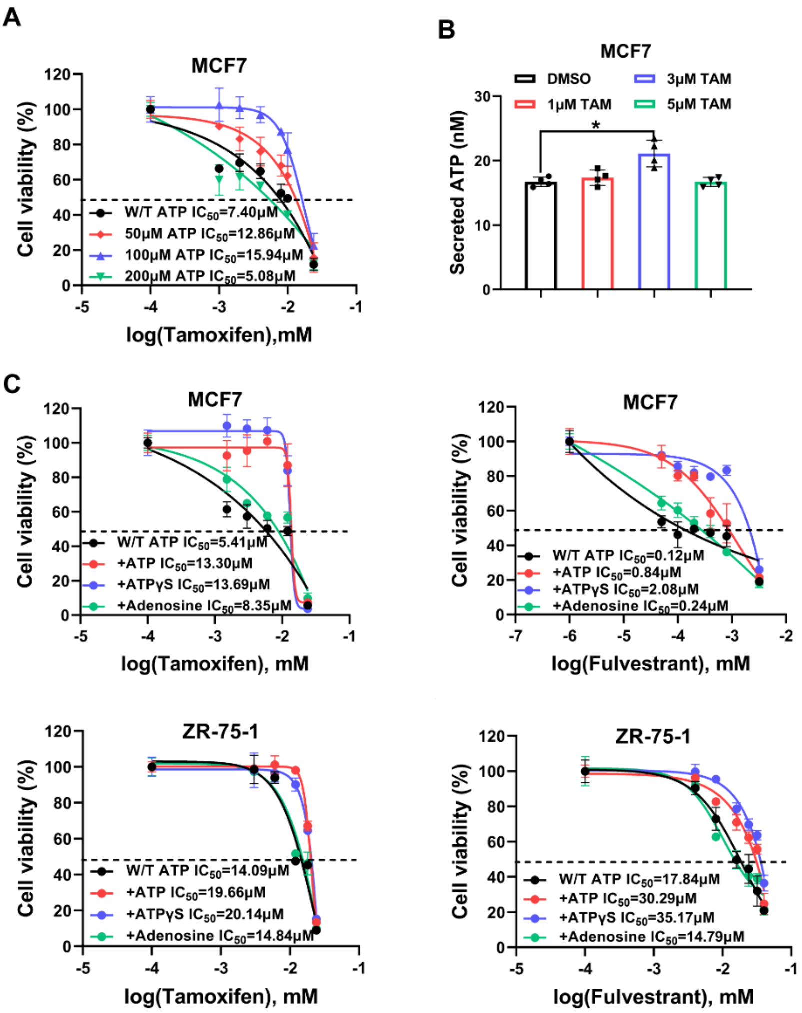

Fig. S1 Extracellular ATP promotes endocrine resistance. (**A**) CCK-8 assay, measured by absorbance at OD450, showed that 100 µM ATP was most effective in promoting tamoxifen resistance in MCF7 cells (n=3). (**B**) ATP secretion from MCF7 cells following tamoxifen treatment (n=4). (**C**) Dose-response curves with increasing doses of tamoxifen or fulvestrant in MCF7 and ZR-75-1 cells treated with or without ATP (100 µM), ATPγS (100 µM), adenosine (100 µM). For each curve, the result for a vehicle-treated control group was set to 100%. Data are representative of at least three independent experiments. Two-tailed unpaired t-test, one-way and two-way analysis of variance (ANOVA). Error bars represent SD; *$p < 0.05$.

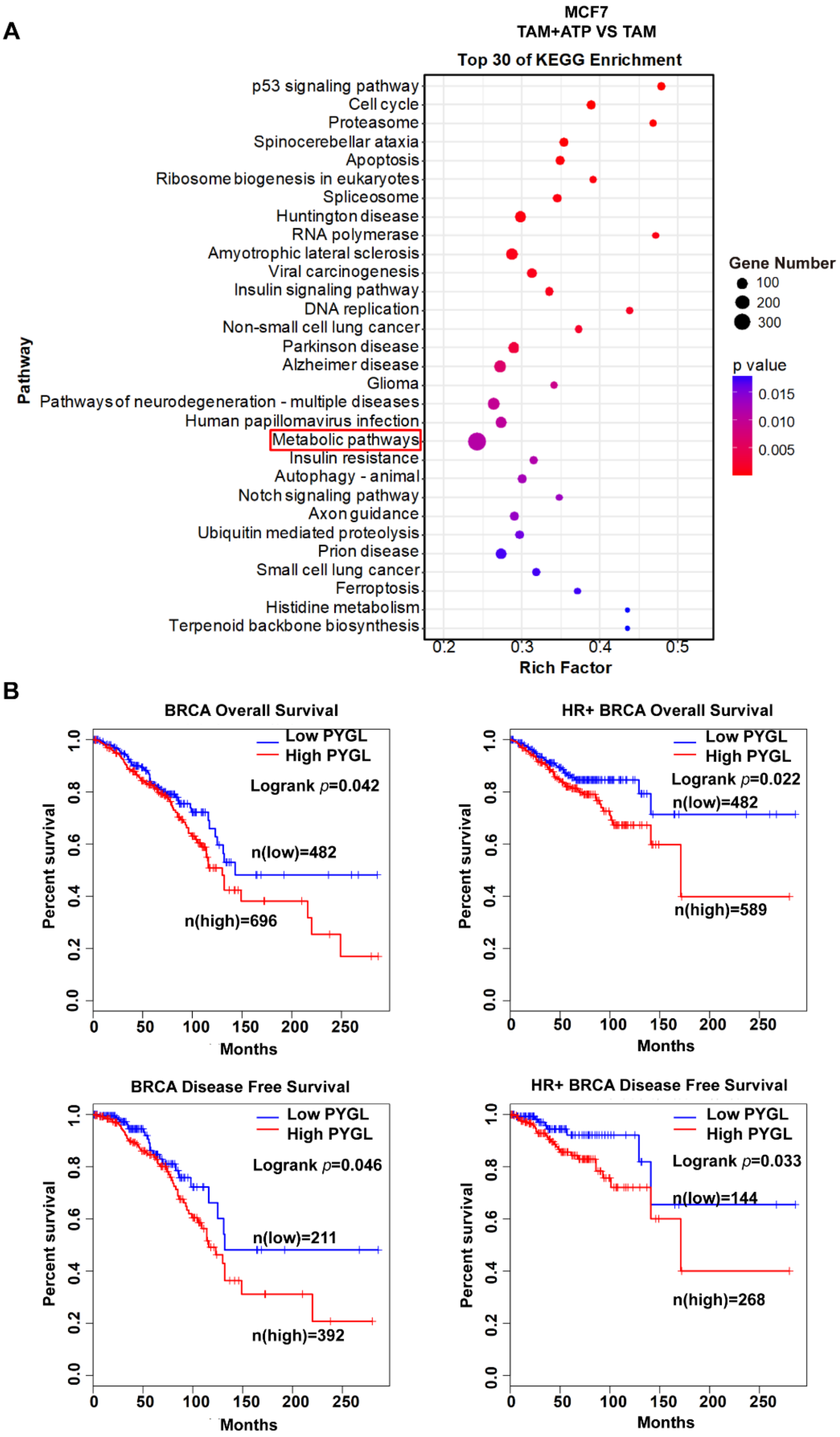
A
MCF7
TAM+ATP VS TAM
Top 30 of KEGG Enrichment
Pathway
p53 signaling pathway
Cell cycle
Proteasome
Spinocerebellar ataxia
Apoptosis
Ribosome biogenesis in eukaryotes
Spliceosome
Huntington disease
RNA polymerase
Amyotrophic lateral sclerosis
Viral carcinogenesis
Insulin signaling pathway
DNA replication
Non-small cell lung cancer
Parkinson disease
Alzheimer disease
Glioma
Pathways of neurodegeneration - multiple diseases
Human papillomavirus infection
Metabolic pathways
Insulin resistance
Autophagy - animal
Notch signaling pathway
Axon guidance
Ubiquitin mediated proteolysis
Prion disease
Small cell lung cancer
Ferroptosis
Histidine metabolism
Terpenoid backbone biosynthesis
0.2 0.3 0.4 0.5
Rich Factor
Gene Number
100
200
300
p value
0.015
0.010
0.005
B
BRCA Overall Survival
Low PYGL
High PYGL
Logrank p=0.042
n(low)=482
n(high)=696
Percent survival
Months
HR+ BRCA Overall Survival
Low PYGL
High PYGL
Logrank p=0.022
n(low)=482
n(high)=589
BRCA Disease Free Survival
Low PYGL
High PYGL
Logrank p=0.046
n(low)=211
n(high)=392
HR+ BRCA Disease Free Survival
Low PYGL
High PYGL
Logrank p=0.033
n(low)=144
n(high)=268
0.0 0.2 0.4 0.6 0.8 1.0
0 50 100 150 200 250

Fig. S2 Extracellular ATP induces upregulation of PYGL in breast cancer cells. (**A**) KEGG enrichment analysis showing the biological processes upon ATP-tamoxifen treatment including metabolic pathway. (**B**) Kaplan-Meier survival analysis (http://gepia.cancer-pku.cn/index.html) showed the negative correlations between survival time of breast cancer patients and expression levels of PYGL in breast cancer and Luminal A&B breast cancer. The mRNA expression level over the median was defined as high expression, and lower than the median was defined as low expression. Long-term survival patterns should be interpreted with caution due to limited information on sequential endocrine therapies.

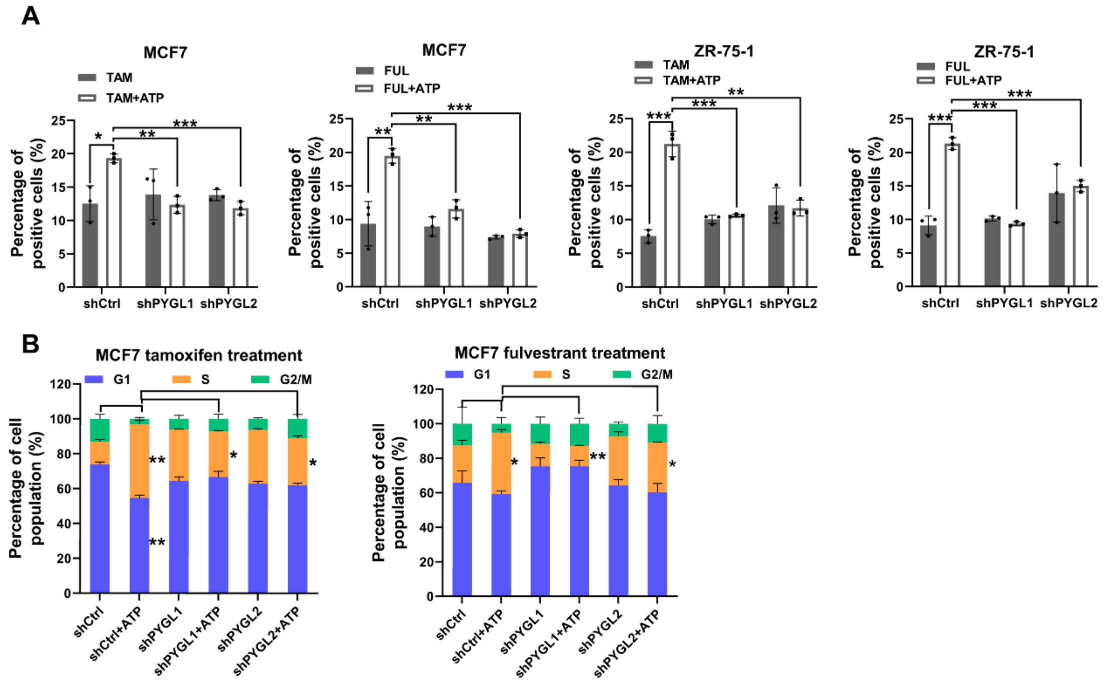

Fig. S3 PYGL knockdown attenuates ATP-driven endocrine resistance in ER+ breast cancer cells. (**A**) Knockdown of PYGL in MCF7 and ZR-75-1 cells altered EdU-positive cells (n=3). (**B**) Knockdown of PYGL in MCF7 cells altered the proportion of cells in G1, S and G2/M phase by FACS analysis (n=3). Data are represented as means ± SD. *P* value is determined by t-test (two-sided). *$p < 0.05$; **$p < 0.01$; ***$p < 0.001$

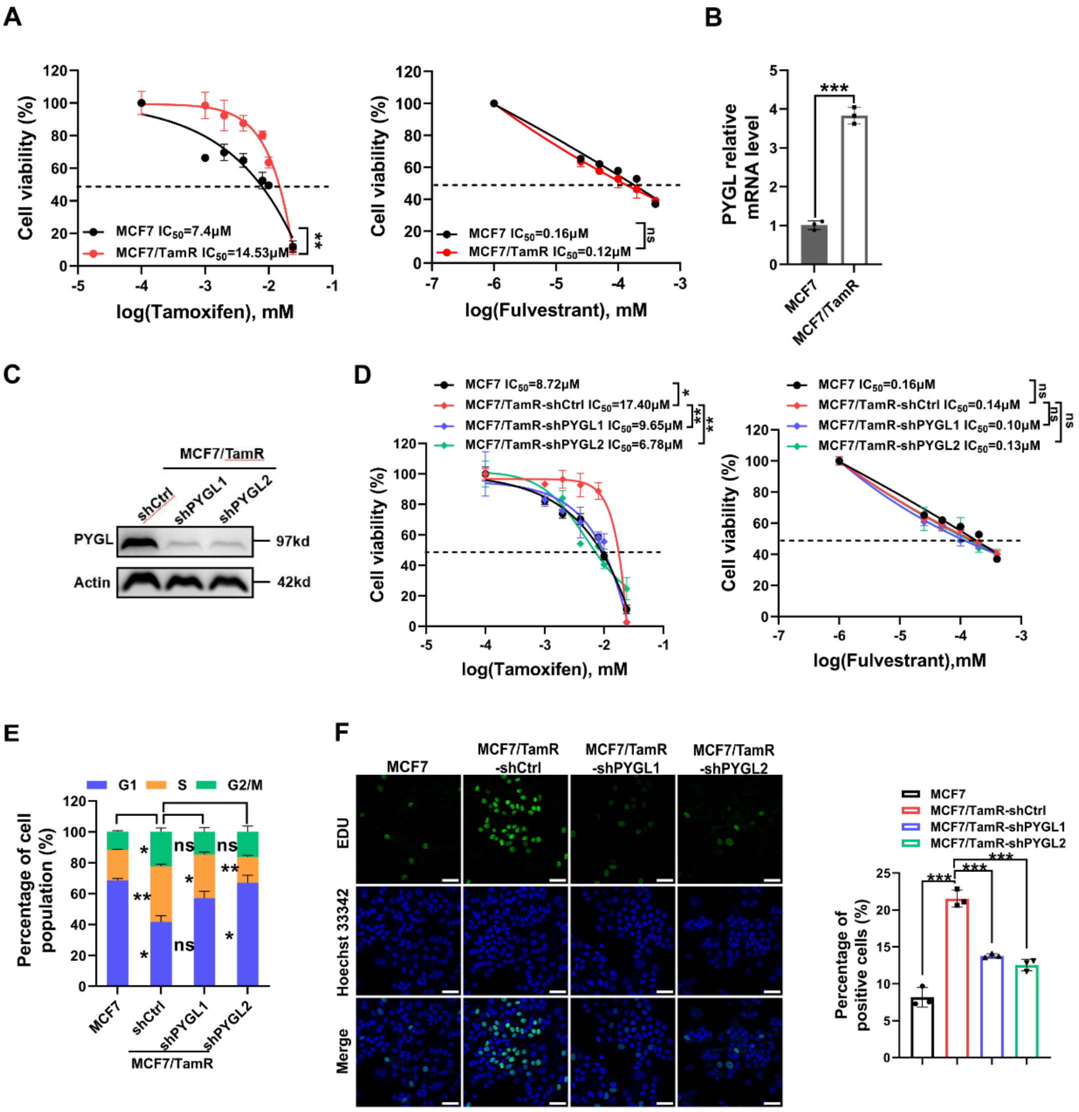

Fig. S4 PYGL is involved in the transition of ER+ breast cancer cells to tamoxifen resistance. The establishment of tamoxifen-resistant MCF7 cells (MCF7/TamR) by treating BC cells with tamoxifen plus 100μM ATP. (**A**) Dose-response curves for tamoxifen resistant MCF7/TamR cells and the corresponding parental MCF7 cells (n=3). (**B**) RT-qPCR analysis of mRNA levels of PYGL in MCF7/TamR cells (n=3). (**C**) Western blot analysis of PYGL knockdown efficiency in MCF7/TamR. (**D**) CCK-8 assay measured by absorbance OD450 showed that PYGL-knockdown altered the cell viability (n=3). (**E**) FACS analysis of the proportion of cells in the G1, S and G2/M phases with MCF7/TamR subjected to PYGL-knockdown (n=3). (**F**) The number of dividing cells in MCF7/TamR-shCtrl and MCF7/TamR-shPYGL cells was quantified by EdU staining (n=3). Scale bars, 50 μm. Data are representative of three independent experiments. Two-tailed unpaired t-test, one-way and two-way analysis of variance (ANOVA). *$p < 0.05$; **$p < 0.01$; ***$p < 0.001$; ns not significant.

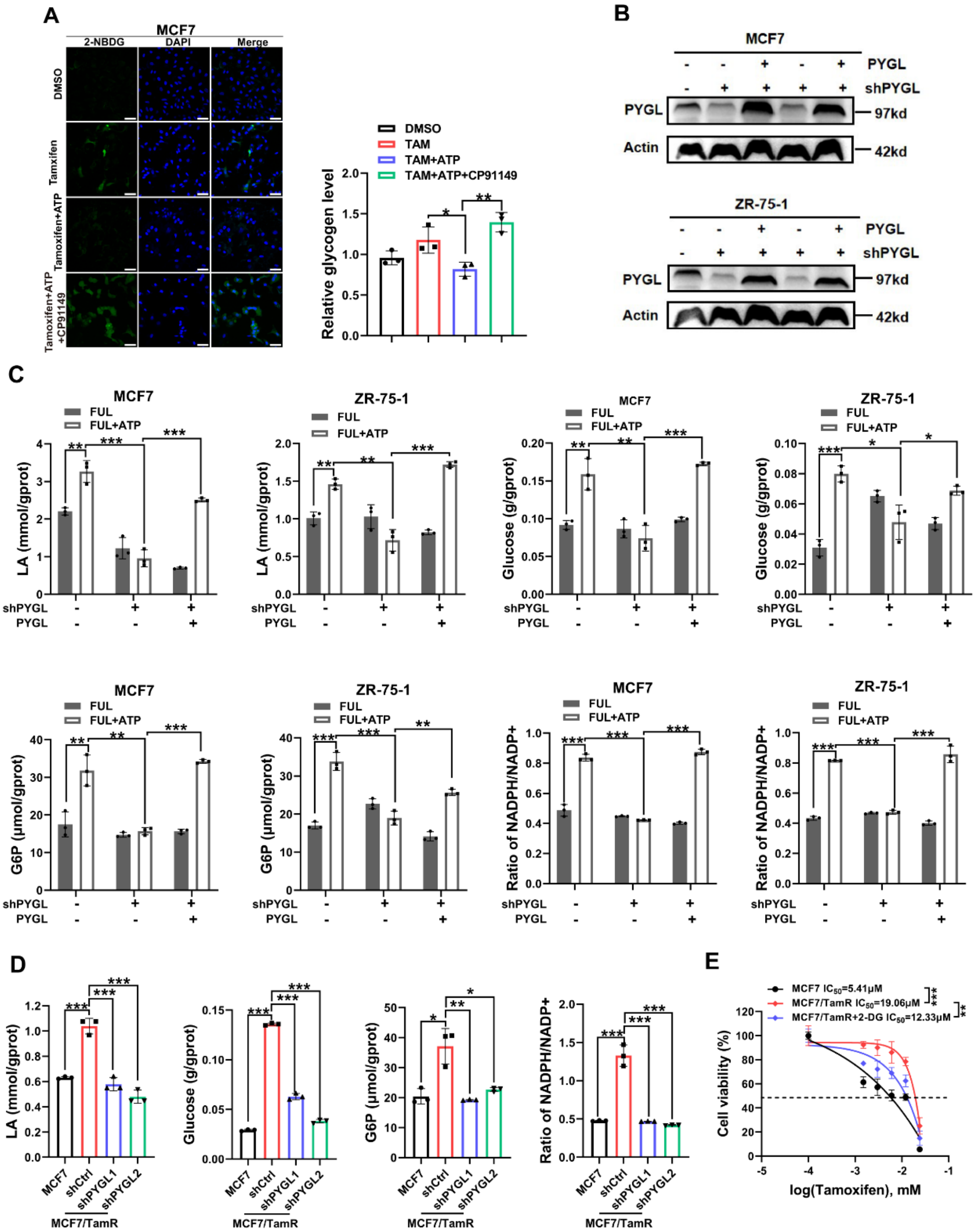

A
MCF7
2-NBDG
DAPI
Merge
DMSO
Tamxifen
Tamoxifen+ATP
Tamoxifen+ATP +CP91149
DMSO
TAM
TAM+ATP
TAM+ATP+CP91149
Relative glycogen level
B
MCF7
PYGL
shPYGL
PYGL
Actin
97kd
42kd
ZR-75-1
C
MCF7
ZR-75-1
FUL
FUL+ATP
LA (mmol/gprot)
Glucose (g/gprot)
G6P (μmol/gprot)
Ratio of NADPH/NADP+
shPYGL
PYGL
D
MCF7
shCtrl
shPYGL1
shPYGL2
MCF7/TamR
E
MCF7 IC50=5.41μM
MCF7/TamR IC50=19.06μM
MCF7/TamR+2-DG IC50=12.33μM
Cell viability (%)
log(Tamoxifen), mM

Fig. S5 The knockdown of PYGL decreases glycolysis. (**A**) Glycogen was visualized by 2-NBDG fluorescence in MCF7 cells after indicated treatment (n=3). Scale bars, 50 μm. (**B**) PYGL-overexpressing cell lines were generated in PYGL-depleted MCF7 and ZR-75-1 cells. (**C**) Knockdown of PYGL attenuated ATP-dependent production of lactate, glucose, G6P and the NADPH/$NADP^+$ ratio in MCF7 and ZR-75-1 cells, whereas PYGL overexpression reversed these effects (n=3). (**D**) Intracellular levels of lactate, glucose, G6P as well as the NADPH/$NADP^+$ ratio, were significantly reduced in PYGL-depleted MCF7/TamR cells (n=3). (**E**) The inhibition of 2-DG on cell glycolysis significantly reduced the resistance to tamoxifen in MCF7/tamR (n=3). Data are representative of at least three independent experiments. Two-tailed unpaired t-test, one-way and two-way analysis of variance (ANOVA). Error bars represent SD; *$p < 0.05$; **$p < 0.01$; ***$p < 0.001$.

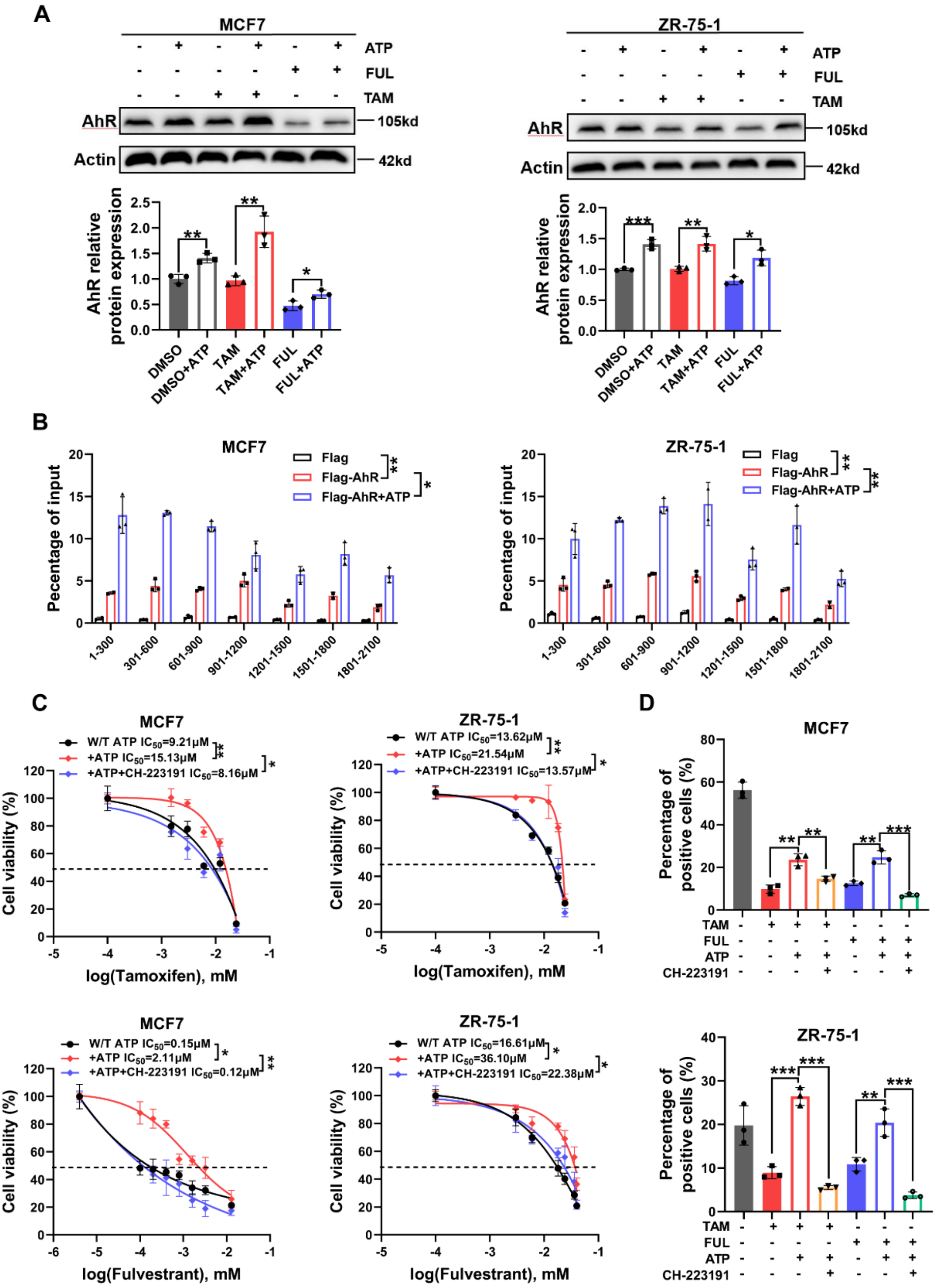
A
MCF7
ATP
FUL
TAM
AhR
105kd
Actin
42kd
AhR relative protein expression
DMSO
DMSO+ATP
TAM
TAM+ATP
FUL
FUL+ATP
ZR-75-1
B
MCF7
Flag
Flag-AhR
Flag-AhR+ATP
Pecentage of input
1-300
301-600
601-900
901-1200
1201-1500
1501-1800
1801-2100
ZR-75-1
C
MCF7
W/T ATP IC50=9.21μM
+ATP IC50=15.13μM
+ATP+CH-223191 IC50=8.16μM
Cell viability (%)
log(Tamoxifen), mM
ZR-75-1
W/T ATP IC50=13.62μM
+ATP IC50=21.54μM
+ATP+CH-223191 IC50=13.57μM
MCF7
W/T ATP IC50=0.15μM
+ATP IC50=2.11μM
+ATP+CH-223191 IC50=0.12μM
log(Fulvestrant), mM
ZR-75-1
W/T ATP IC50=16.61μM
+ATP IC50=36.10μM
+ATP+CH-223191 IC50=22.38μM
D
MCF7
Percentage of positive cells (%)
TAM
FUL
ATP
CH-223191
ZR-75-1

Fig. S6 ATP promotes endocrine resistance via the AhR-PYGL signaling axis. (**A**) Expressions of AhR protein after 6 days of DMSO, tamoxifen, fulvestrant with or without ATP treatment were examined by western blot (n=3). (**B**) Flag or Flag-AhR were transfected into MCF7 and ZR-75-1 cells. Different promoter regions of PYGL pulled down with the Flag antibody were analyzed by RT-qPCR (n=3). (**C**) CH-223191, as a selective AhR antagonist reversed the ability of ATP to promote endocrine resistance in ER+ BC cells (n=3). (**D**) The number of dividing cells in MCF7 and ZR-75-1 cells was quantified by EdU staining (n=3). Data are representative of at least three independent experiments. Two-tailed unpaired t-test, one-way and two-way analysis of variance (ANOVA). Error bars represent SD; *$p < 0.05$; **$p < 0.01$; ***$p < 0.001$.

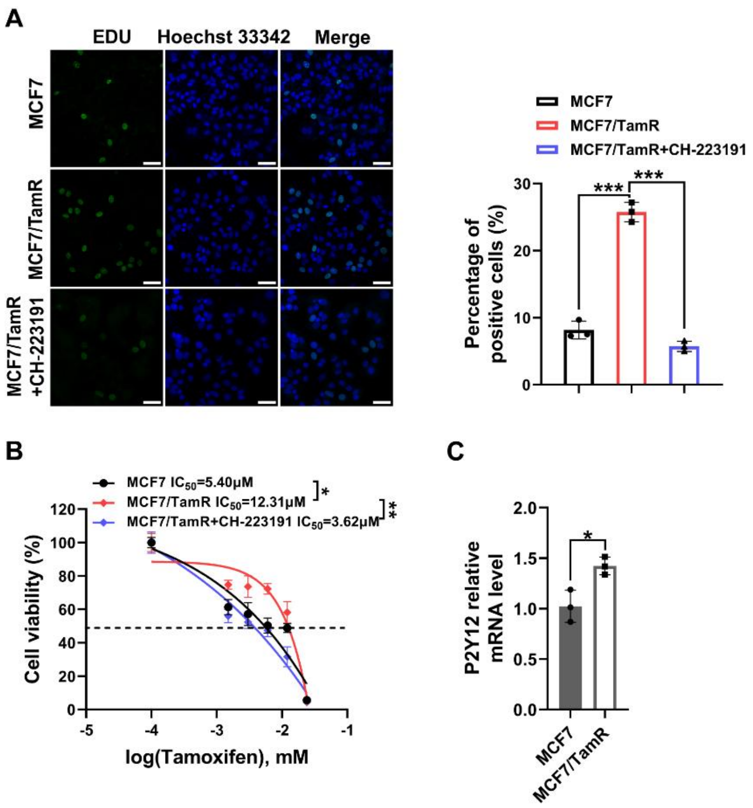

Fig. S7 Inhibition of AhR enhances the sensitivity of MCF7/TamR cells. (**A**) The number of dividing cells in MCF7, MCF7/TamR and MCF7/TamR cells subjected to CH-223191 were measured by EdU staining (n=3). Scale bars, 50 μm. (**B**) The inhibition of CH-223191 on AhR signaling significantly reduced the resistance to tamoxifen in MCF7/TamR (n=3). (**C**) RT-qPCR analysis of P2Y12 expression in MCF7/TamR cells (n=3). Data are representative of at least three independent experiments. Two-tailed unpaired t-test, one-way and two-way analysis of variance (ANOVA). Error bars represent SD; *$p < 0.05$; **$p < 0.01$; ***$p < 0.001$.

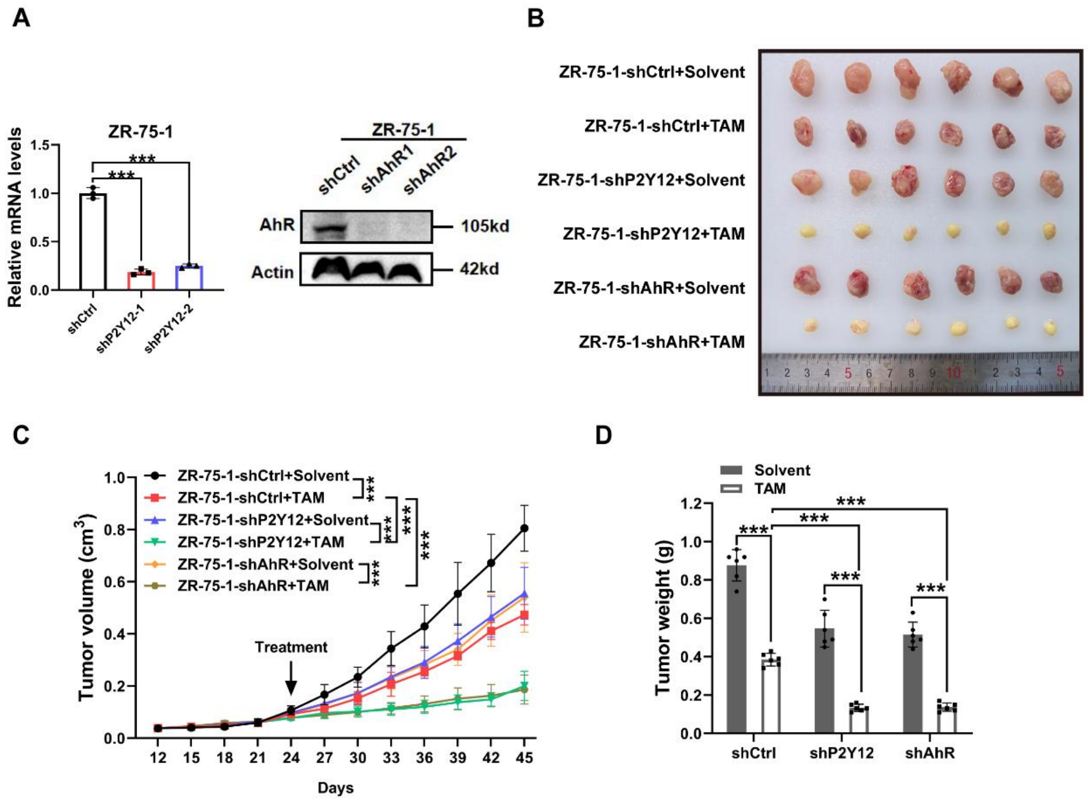

Fig. S8 P2Y12 and AhR promotes xenograft tumor tamoxifen resistance in vivo. Stably transfected ZR-75-1 cells (shCtrl, shP2Y12, shAhR) were subcutaneously inoculated into 5-week-old female Balb/c mice (n = 6), followed by tamoxifen treatment once the mean tumor volume reached approximately 100 mm³. (**A**) P2Y12- and AhR-knockdown ZR-75-1 cell lines were generated. (**B**) Bright-field images of excised subcutaneous tumors. (**C**) Primary tumor volumes were measured and quantified at 3-day intervals. (**D**) Weights of excised primary tumors at the experimental endpoint. Two-tailed unpaired t-test, one-way and two-way analysis of variance (ANOVA). Error bars represent SD; ****p* < 0.001.

Table S1: Differential genes obtained by ATP treatment in MCF7 cells for RNA-seq (|log2 FC|≥0.6, q≤0.04).

Gene_id	Gene name	log2FoldChange	q. adj	level
ENSG00000115461	IGFBP5	-2.072145389	3.68E-22	down
ENSG00000196620	UGT2B15	-1.908528718	0.027099068	down
ENSG00000145824	CXCL14	-1.754667928	6.29E-08	down
ENSG00000135063	FAM189A2	-1.627195533	1.49E-08	down
ENSG00000164742	ADCY1	-1.569379134	1.82E-11	down
ENSG00000185052	SLC24A3	-1.553898363	6.92E-07	down
ENSG00000175356	SCUBE2	-1.552993421	2.11E-05	down
ENSG00000183401	CCDC159	-1.497909856	2.59E-04	down
ENSG00000107984	DKK1	-1.397903349	1.04E-06	down
ENSG00000139352	ASCL1	-1.395276296	2.23E-10	down
ENSG00000106541	AGR2	-1.394933586	0.011017961	down
ENSG00000169136	ATF5	-1.39049972	0.015369333	down
ENSG00000102683	SGCG	-1.35925731	3.65E-09	down
ENSG00000244242	IFITM10	-1.291184681	0.025602916	down
ENSG00000158406	H4C8	-1.217945722	0.002887274	down
ENSG00000111328	CDK2AP1	-0.701438712	0.018868183	down
ENSG00000134107	BHLHE40	0.684002747	0.032355863	up
ENSG00000187867	PALM3	1.196096296	1.00E-04	up
ENSG00000100504	PYGL	1.19676355	3.46E-07	up
ENSG00000164932	CTHRC1	1.213495849	0.001159835	up
ENSG00000164039	BDH2	1.24019904	4.97E-05	up
ENSG00000156804	FBXO32	1.272884162	2.13E-05	up
ENSG00000185215	TNFAIP2	1.2840221	1.70E-07	up
ENSG00000144063	MALL	1.310213491	0.01645307	up
ENSG00000164403	SHROOM1	1.334730021	1.95E-05	up
ENSG00000176945	MUC20	1.423584672	0.001727211	up
ENSG00000196878	LAMB3	1.45632801	0.001583837	up
ENSG00000147689	FAM83A	1.464604375	0.014348693	up
ENSG00000064787	BCAS1	1.506116488	0.014752849	up
ENSG00000130066	SAT1	1.525771925	4.81E-05	up
ENSG00000116016	EPAS1	1.569889126	7.14E-15	up
ENSG00000137868	STRA6	1.58861451	1.57E-06	up
ENSG00000120708	TGFBI	1.590350902	0.003680996	up
ENSG00000188488	SERPINA5	1.688519457	0.003122298	up
ENSG00000198183	BPIFA1	1.693412078	0.001021835	up
ENSG00000125999	BPIFB1	1.694656404	6.60E-05	up
ENSG00000170899	GSTA4	1.708874153	1.04E-06	up
ENSG00000086548	CEACAM6	1.713133934	9.37E-04	up
ENSG00000105641	SLC5A5	1.74272666	9.99E-10	up
ENSG00000002726	AOC1	1.753960045	0.002661848	up

ENSG00000080031	PTPRH	1.766134993	0.009267266	up
ENSG00000117983	MUC5B	1.804029248	0.002664332	up
ENSG00000165507	DEPP1	1.856747874	0.020688794	up
ENSG00000140465	CYP1A1	1.893280347	8.63E-05	up
ENSG00000105388	CEACAM5	2.020912508	4.85E-14	up
ENSG00000244067	GSTA2	2.155950712	8.23E-09	up
ENSG00000142910	TINAGL1	2.232462626	4.80E-05	up
ENSG00000143556	S100A7	2.380936902	7.79E-04	up
ENSG00000138378	STAT4	2.432380507	6.42E-04	up
ENSG00000184254	ALDH1A3	2.750419545	3.68E-22	up

Table S2: Characteristics and clinical data of 8 patients with ER+ BC for organoid culture.

NO.	Gender	Age	ER[b] (percentage of positive cells)	PR[d] (percentage of positive cells)	[c]HER2 overexpression (IHC score)
BC[a]-1	Female	60	90%	13%	0
BC-2	Female	35	75%	89%	0
BC-3	Female	45	40%	0	0
BC-4	Female	41	30%	5%	1+
BC-5	Female	51	80%	20%	1+
BC-6	Female	50	60%	90%	0
BC-7	Female	54	80%	70%	2+
BC-8	Female	32	90%	90%	1+

[a]BC, Breast cancer; [b]ER, Estrogen receptor; [c]HER2, Human epidermal growth factor receptor 2; [d]PR, Progesterone receptor.

Table S3: Characteristics and clinical data of 34 patients in a clinical cohort.

NO.	Gender	Age	TNM[a] staging	Molecular Subtype	Treatment	Recurrence
Case1	Female	59	T1N1M0	Luminal A	Chemotherapy; Endocrine Therapy	No
Case2	Female	62	T1N0M0	Luminal A	Chemotherapy; Radiation; Endocrine Therapy	No
Case3	Female	46	T1N1M0	Luminal A	Chemotherapy; Radiation; Endocrine Therapy	No
Case4	Female	44	T1N0M0	Luminal A	Chemotherapy; Endocrine Therapy	No
Case5	Female	33	T1N1M0	Luminal B	Chemotherapy; Radiation; Endocrine Therapy	No
Case6	Female	58	T1N1M0	Luminal B	Chemotherapy; Endocrine Therapy	No
Case7	Female	46	T2N3M0	Luminal B	Chemotherapy; Radiation; Endocrine Therapy	No
Case8	Female	53	T1N0M0	Luminal B	Radiation; Endocrine Therapy	No
Case9	Female	60	T1N0M0	Luminal B	Chemotherapy; Endocrine Therapy	No
Case10	Female	54	T1N0M0	Luminal B	Chemotherapy; Endocrine Therapy	No
Case11	Female	66	T1N1M0	Luminal B	Chemotherapy; Radiation; Endocrine Therapy	No
Case12	Female	39	T2-3N0M0	Luminal A	Chemotherapy; Radiation; Endocrine Therapy	No
Case13	Female	42	T1N0M0	Luminal B	Chemotherapy; Radiation; Endocrine Therapy	No
Case14	Female	47	T1N0M0	Luminal A	Chemotherapy; Endocrine Therapy	No
Case15	Female	67	T1N0M0	Luminal A	Chemotherapy; Endocrine Therapy	No
Case16	Female	67	T1N0M0	Luminal B	Chemotherapy; Endocrine Therapy	No
Case17	Female	43	T1N1M0	Luminal B	Chemotherapy; Endocrine Therapy	No
Case18	Female	45	T1N0M0	Luminal B	Chemotherapy; Endocrine Therapy	No
Case19	Female	39	T1N0M0	Luminal A	Radiation; Endocrine Therapy	No
Case20	Female	45	T2N1M0	Luminal B	Chemotherapy; Radiation; Endocrine Therapy	No

Case21	Female	67	T2N0M0	Luminal B	Chemotherapy; Endocrine Therapy	No
Case22	Female	51	T2N0M0	Luminal A	Chemotherapy; Endocrine Therapy	No
Case23	Female	47	T1N0M0	Luminal B	Chemotherapy; Endocrine Therapy	No
Case24	Female	59	T1N0M0	Luminal B	Chemotherapy; Radiation; Endocrine Therapy	No
Case25	Female	67	T2N2M0	Luminal B	Chemotherapy; Radiation; Endocrine Therapy	Yes
Case26	Female	48	T2N0M0	Luminal B	Chemotherapy; Endocrine Therapy	Yes
Case27	Female	44	T1N0M0	Luminal B	Chemotherapy; Radiation; Endocrine Therapy	Yes
Case28	Female	27	T1N1M0	Luminal B	Chemotherapy; Radiation; Endocrine Therapy	Yes
Case29	Female	66	T1N0M0	Luminal A	Radiation; Endocrine Therapy	Yes
Case30	Female	62	T1N2M0	Luminal B	Chemotherapy; Radiation; Endocrine Therapy	Yes
Case31	Female	39	T2N1M0	Luminal B	Chemotherapy; Radiation; Endocrine Therapy	Yes
Case32	Female	40	T2N1M0	Luminal A	Chemotherapy; Radiation; Endocrine Therapy	Yes
Case33	Female	32	T1N1M0	Luminal A	Chemotherapy; Radiation; Endocrine Therapy	Yes
Case34	Female	47	T2N1M0	Luminal B	Chemotherapy; Radiation; Endocrine Therapy	Yes

[a]TNM, Tumor Node Metastasis

Supplementary methods

Lentiviral transduction. The PYGL-shRNAs (shPYGL) and negative control shRNAs (shCtrl) were purchased from Shanghai GeneChem Co. (Shanghai, China). Lentiviral particles (titer: 1×10^9 TU/mL) were stored at -80°C prior to use. Cells were seeded in 6-well plates ($6–10 \times 10^4$ cells/well) and cultured for 16–24 h. Lentivirus was then added to the culture medium and incubated for 16 hours. After incubation, the medium was replaced with fresh complete medium. Stably transduced cells were selected by continuous treatment with 1 μg/mL puromycin for 72 h.

Sequences of the shRNAs used in this study

Gene name	Target Sequences
PYGL sh-RNA#1	TACCAGCTTGGATTGGATATA
PYGL sh-RNA#2	CCTATGTCAAGTGTCAAGATA

RNA extraction and real-time RT-PCR. Total RNA was isolated using Trizol reagent (Invitrogen) following the manufacturer's protocol. One microgram of RNA was reverse transcribed using the StarScript II RT MasterMix (Genstar). Real-time qPCR was conducted using SYBR qPCR Mix (TOYOBO, Osaka, Japan) on the CFX96 Real-Time PCR Detection System (Bio-Rad, Hercules, CA, USA). Relative mRNA expression levels were calculated using the $2^{-\triangle\triangle CT}$ method. All qPCR reactions were performed in triplicate. Primer sequences are listed below.

Primers used in this study

Gene name	Forward primer	Reverse primer
GAPDH	GTCTCCTCTGACTTCAACAGCG	ACCACCCTGTTGCTGTAGCCAA
UGT2B15	ACTTTAGGTTCCAATACTCGACTG	CGCCTCATAGATGCCATTGGTTC
ADCY1	TCGCGTCAACAGGTACATCAGC	CGCTGGTGAAATACTCGTCCTG
PYGL	CACTTCAGTGGCAGATGTGGTG	GCAGTGGAAATCTGCTCTGACAG
BDH2	CACAACCAAGGCAGCCGTGATT	CTTGTAGAGATGGCGTATCAACTG
SAT1	TACCACTGCCTGGTTGCAGAAG	CTTGCCAATCCACGGGTCATAG
GSTA4	ACAGACCCGAAGCATTCTCCAC	AGTTCCAGCAGATCCAGTGTCC
AOC1	TACGTCCACGCCACCTTCTACA	GTCTGGAAGCTGTTCTTGGTGC
CYP1A1	GATTGAGCACTGTCAGGAGAAGC	ATGAGGCTCCAGGAGATAGCAG
GSTA2	CTGCCCTTTAGTCAACCTGAGG	ACAAGGTAGTCTTGTCCGTGGC
ALDH1A3	CTGCTACAACGCCCTCTATGCA	GTCGCCAAGTTTGATGGTGACAG
PYGB	CCAGACTGCTTCAAGGACATCG	GTTCCTGATGACCTTCTTGGTCC
PYGM	AGGCTGTGTTGGACCGAAACCT	AAGCGACGGATGATGTCCTGGA
BHLHE40	TAAAGCGGAGCGAGGACAGCAA	GATGTTCGGGTAGGAGATCCTTC
JUN	CCTTGAAAGCTCAGAACTCGGAG	TGCTGCGTTAGCATGAGTTGGC
AhR	GTCGTCTAAGGTGTCTGCTGGA	CGCAAACAAAGCCAACTGAGGTG
STAT4	CAGTGAAAGCCATCTCGGAGGA	TGTAGTCTCGCAGGATGTCAGC
IRF2	TAGAGGTGACCACTGAGAGCGA	CTCTTCATCGCTGGGCACACTA
ELF3	CATGACCTACGAGAAGCTGAGC	GACTCTGGAGAACCTCTTCCTC

Gene name	Forward primer	Reverse primer
FOSL1	GGAGGAAGGAACTGACCGACTT	CTCTAGGCGCTCCTTCTGCTTC
HNF4G	GAGGTTTGGAGAGTTGCTTCTGC	GATGACTGCCATCATTGGAAGCC
P2X1	CCTCATCAGCAGTGTCTCTGTG	CATGACCACGAAGGAGTTGTCC
P2X2	CATCGTGCAGAAAAGCTACCAGG	CCTGGTGATGATGCTGAACACG
P2X3	GCGTTTCTGAGAAAAGCAGCGTG	CGGATGCCAAAAGCCTTCAGGA
P2X4	GTGGCGGATTATGTGATACCAGC	CACACAGTGGTCGCATCTGGAA
P2X5	CGCTTTGACGTGATGGTGAACG	TCCTGGGAACTGTCTTCTAGGC
P2X6	AAGCACTGCCGCTATGAACCAC	CCAGTGAACTCTGATGCCTACAG
P2X7	CGACTAGGAGACATCTTCCGAG	GCAGTGATGGAACCAACGGTCT
P2Y1	GCCATCTGGATGTTCGTCTTCC	TGGCAGAGTCAGCACGTACAAG
P2Y2	CGAGGACTTCAAGTACGTGCTG	GTGGACGCATTCCAGGTCTTGA
P2Y11	CCCATACTGGTGGTTGAGTTCC	AGCTGGACAGAGAAGACCACGG
P2Y12	TGCCAAACTGGGAACAGGACCA	TGGTGGTCTTCTGGTAGCGATC
P2Y13	GCCGACTTGATAATGACACTCATG	CCTAACAGCACGATGCCCACAT
PANX1	CCTGAGAAACGACAGCACC	TGTAGACAACCACGGGAGC
PANX2	CCCAGAGCCAGGGAAGAG	GGCGACAAGGAGAAAGTGC
PANX3	TCATCATCAGCGAACTGG	CGTTCTTTCCGAGCCTT
CDK1	GGAAACCAGGAAGCCTAGCATC	GGATGATTCAGTGCCATTTTGCC
CDK4	CCATCAGCACAGTTCGTGAGGT	TCAGTTCGGGATGTGGCACAGA
Cyclin D1	TCTACACCGACAACTCCATCCG	TCTGGCATTTTGGAGAGGAAGTG
Cyclin E1	TGTGTCCTGGATGTTGACTGCC	CTCTATGTCGCACCACTGATACC
Rb	CAGAAGGTCTGCCAACACCAAC	TTGAGCACACGGTCGCTGTTAC
Negative	CTAAGGACGAGATGCACATGG	TCAAGTTTCCAACTCCAACAGG
1~300	GGCGGGCCCCAAACTTTCGTGC	GGTCCGTCAGGGGCTTCGCCATG
301~600	GGCTGGCAAGTGAGGTTGGGACA	CGTCGTTGGCAGACTGCTGTTTT
601~900	AAGCCCCTGCCGCATAATGACCC	ACCAAAGTTCGCCCCGCTCGTAG
901~1200	AACTTAATTCTGGCAATGGACTG	CTTAAACCTTGGTAAAGGCAAAC
1201~1500	ATCCTGGCTAACATGGTGAAACC	CCTCCTGAATAGCTGGGACTACA
1501~1800	GGGCGGATCACAAGGTCAGGAGA	TTACCCAGGCAGAGTGCAGTGGC
1801~2100	CAAACTTCAGAATCTGGGAGGAC	GATTCAGACCAAATGTGACCCTC

Chromatin immunoprecipitation. HEK293T cells were transiently transfected with pcDNA3.1-FLAG-AhR, pcDNA3.1-FLAG-ELF3, pcDNA3.1-FLAG-HNF4G, or pcDNA3.1-FLAG. After 48 hours of transfection, cells were cross-linked with 0.75% formaldehyde for 10 minutes at room temperature. Glycine was used to quench the cross-linking, and samples were washed with ice-cold PBS, collected, and incubated in ChIP lysis buffer containing protease inhibitors for 20 minutes on ice. Following sonication, the sheared, cross-linked chromatin was harvested. Chromatin extracts

containing DNA fragments ranging from 200 to 1000 base pairs were immunoprecipitated using an anti-FLAG antibody. Chromatin immunoprecipitation samples were analyzed by RT-qPCR. Primer sequences are listed above.

Western blot analysis. Cells were collected and resuspended in RIPA Lysis Buffer (APPLYGEN). Lysates were separated by SDS-PAGE and transferred onto a polyvinylidene fluoride (PVDF) membrane. Membranes were blocked with 5% nonfat milk for 1 h. Primary antibodies were applied overnight at 4°C (for antibody details, see the list above). HRP-conjugated secondary antibodies, goat anti-Rabbit IgG (ZB2301) or goat anti-mouse IgG (H+L) (ZB2305), were diluted at 1:4000. Western blots were quantified using ImageJ (NIH, USA).

Antibody used in this study

Antibody	Company	Catalogue number	Application
Anti-β actin	ZSGB-BIO	TA-09	1:2000 (WB)
Anti-PYGL	Proteintech	15851-1-AP-50UL	1:1000 (WB), 1:1000 (IHC)
Anti- ERα	Abcam	Ab32063	1:500 (IHC)
Anti-AhR	Proteintech	28727-1-AP	1:1000 (WB), 1:500 (IHC)
Anti-P2Y12	Abcam	ab254347	1:1000 (IHC)
Anti-ki67	GTR	GM724002	Ready-to-use
Anti-c-caspase3	CST	9661T	1:400 (IHC)

H&E staining and immunohistochemical staining. For histological examination, 4-μm sections were stained with hematoxylin and eosin (H&E) and subjected to immunohistochemical (IHC) staining using standard protocols. Primary antibodies used are listed above. Data were collected from approximately five random areas per section, and images were analyzed using ImageJ software (NIH, Bethesda, MD, USA).

Lactate, Glucose, ATP, Glucose-6-Phosphate and NADPH/NADP$^+$ Ratio Assays. Lactate levels were measured using the L-Lactic Acid (LA) Colorimetric Assay Kit (Elabscience) according to the manufacturer's instructions. Briefly, cells were homogenized in 200 μL of ice-cold PBS, centrifuged at 10,000 g for 10 minutes, and the supernatant was assayed for lactate content.

Glucose production was measured using the Glucose (Glu) Colorimetric Assay Kit (Elabscience). Cells were homogenized in 200 μL of ice-cold PBS, boiled for 10 minutes, and centrifuged at 10,000 g for 10 minutes. The supernatant was analyzed for glucose content.

ATP levels were determined using the ATP Assay Kit (Beyotime) following the manufacturer's protocol. Briefly, cells were lysed with 200 μL of lysis buffer, centrifuged at 12,000 g for 5 minutes at 4°C, and ATP levels in the supernatant were measured.

Glucose-6-phosphate (G6P) levels were assessed using the Glucose-6-Phosphate (G6P) Colorimetric Assay Kit (Elabscience). Cells were lysed with 200 μL of extracting solution, centrifuged

at 10,000 g for 10 minutes at 4°C, and G6P levels in the supernatant were measured.

The NADP+/NADPH ratio was determined using the NADP+/NADPH Colorimetric Assay Kit (Elabscience). Briefly, cells were lysed with 800 µL of extracting solution, centrifuged at 12,000 g for 10 minutes at 4°C, and the supernatant was analyzed for NADP+ and NADPH levels. The results were normalized to protein content or cell number.

Glycogen staining. Two methods were used to label glycogen in this study:

(i) Periodic Acid-Schiff (PAS) Staining: Tumor slides were deparaffinized, rehydrated, and treated with periodic acid (Solarbio) for 5-10 minutes at room temperature. After rinsing, the slides were incubated in Schiff's reagent for 10-15 minutes, washed in running water for 5 minutes, and counterstained with hematoxylin.

(ii) Fluorescent Labeling with 2-NBDG: To label glycogen in live breast cancer cells, 2-NBDG, a fluorescent glucose analog, was used. Cells were seeded in 24-well confocal dishes and incubated overnight in glucose-free DMEM (Meilunbio). The next day, cells were treated with 100 µM 2-NBDG (MedChemExpress, HY-116215) in glucose-free media for 2 h. Fluorescence was imaged using a confocal microscope (LSM 700, Zeiss, Oberkochen, Germany).

RNA interference. siRNA oligonucleotides targeting human AhR, ELF3, HNF4G, STAT4, P2X4, P2X5, P2Y2, and P2Y12 were synthesized by Tsingke and transfected using Lipofectamine RNAiMAX reagent.

Sequences of the siRNAs used in this study

Gene name	sense（5'-3'）	antisense（5'-3'）
AHR si-RNA#1	AUCCACAGUCAGCCAUAAUAATT	UUAUUAUGGCUGACUGUGGAUTT
AHR si-RNA#2	CGGCAUAGAGACCGACUUAAUTT	AUUAAGUCGGUCUCUAUGCCGTT
ELF3 si-RNA#1	GCUCUUCUGAUGAGCUCAGUUTT	AACUGAGCUCAUCAGAAGAGCTT
ELF3 si-RNA#2	CAACUACUUCAGUGCGAUGUATT	UACAUCGCACUGAAGUAGUUGTT
HNF4G si-RNA#1	ACAUCAAUGAUCGGCAGUAUGTT	CAUACUGCCGAUCAUUGAUGUTT
HNF4G si-RNA#2	GCAUUCGUAAGAGUCACGUUUTT	AAACGUGACUCUUACGAAUGCTT
STAT4 si-RNA#1	GCGAGACUACAAAGUUAUUAUTT	AUAAUAACUUUGUAGUCUCGCTT
STAT4 si-RNA#2	CGUGGUCUUAACUCAGAUCAATT	UUGAUCUGAGUUAAGACCACGTT
P2X4 si-RNA	GUCGUGCAUUUAUGAUGCUAATT	UUAGCAUCAUAAAUGCACGACTT
P2X5 si-RNA	CGUGACAAGAAGUACGAGGAATT	UUCCUCGUACUUCUUGUCACGTT
P2Y2 si-RNA	CCAUCAACAUGGCCUACAATT	UUGUAGGCCAUGUUGAUGGTT
P2Y12 si-RNA	CCUUGAACGCCUGCCUUGAUCCAU UTT	AAUGGAUCAAGGCAGGCGUUCAAG GTT
