# Supplementary figures and images for: Extracellular ATP promotes endocrine resistance in ER+ breast cancer through upregulation of PYGL
Source: Cell Death Dis. 2026 Apr 13;17(1):476. doi: 10.1038/s41419-026-08736-8 (PMC13184142; doi:10.1038/s41419-026-08736-8)

Fig 2C

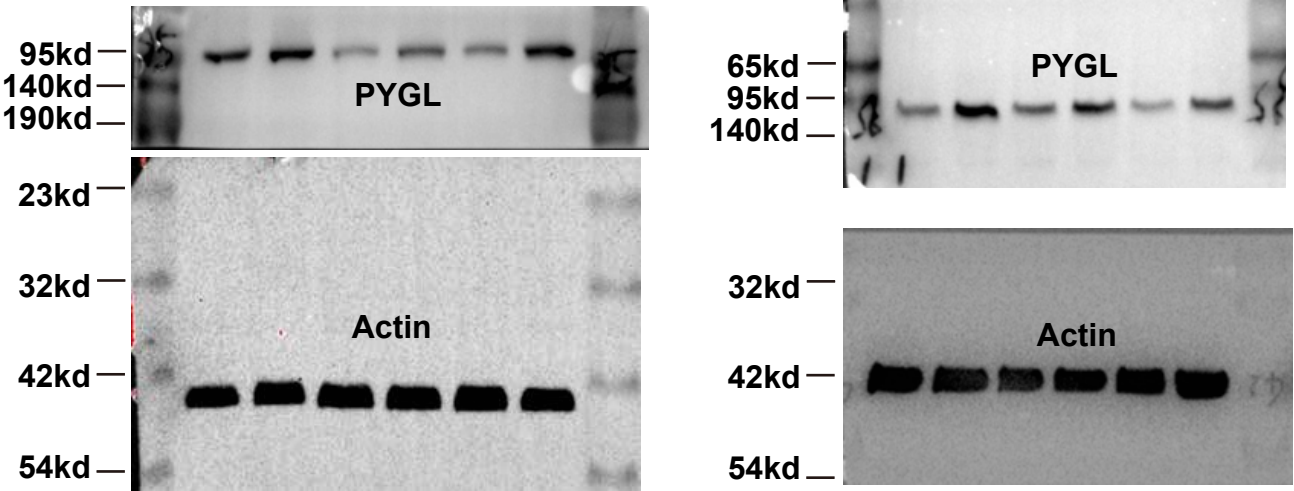

Fig 2D

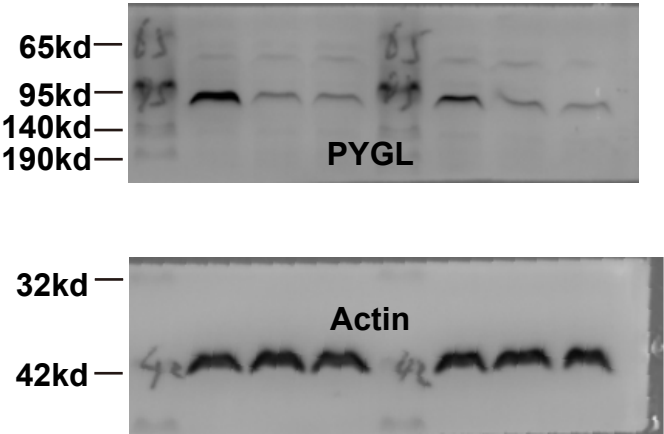

Fig 4C

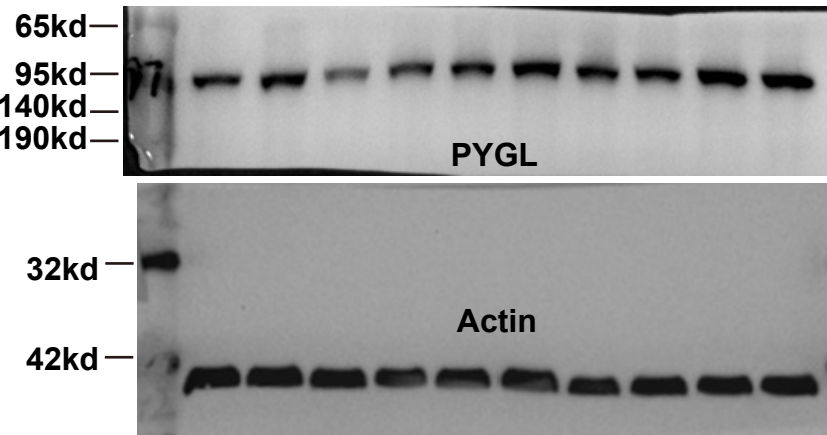

Fig 4G

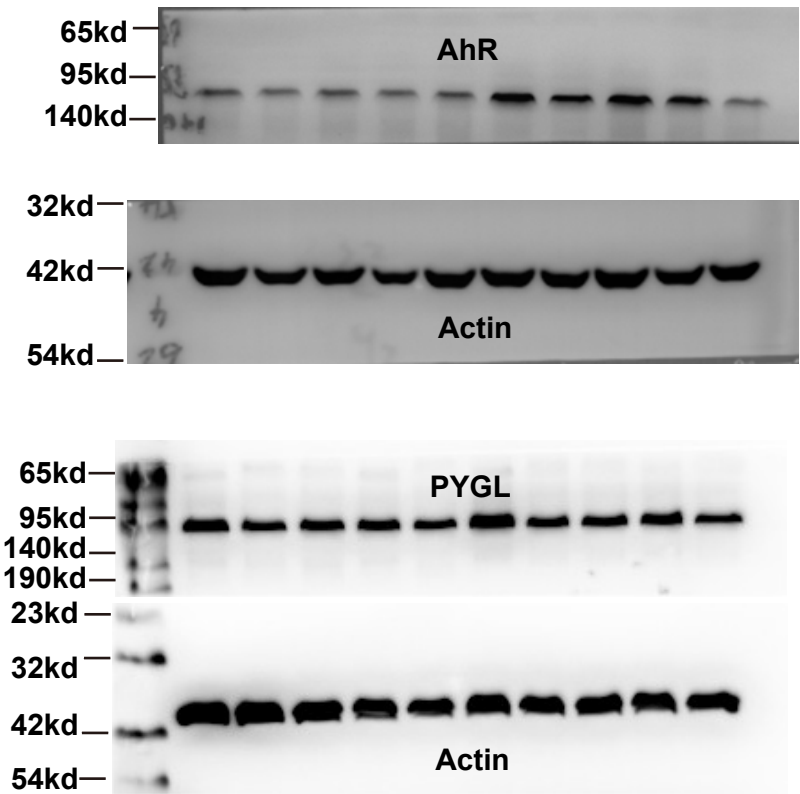

**Fig S4C**

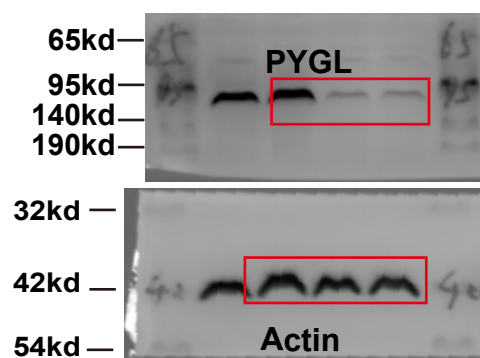

**Fig S5B**

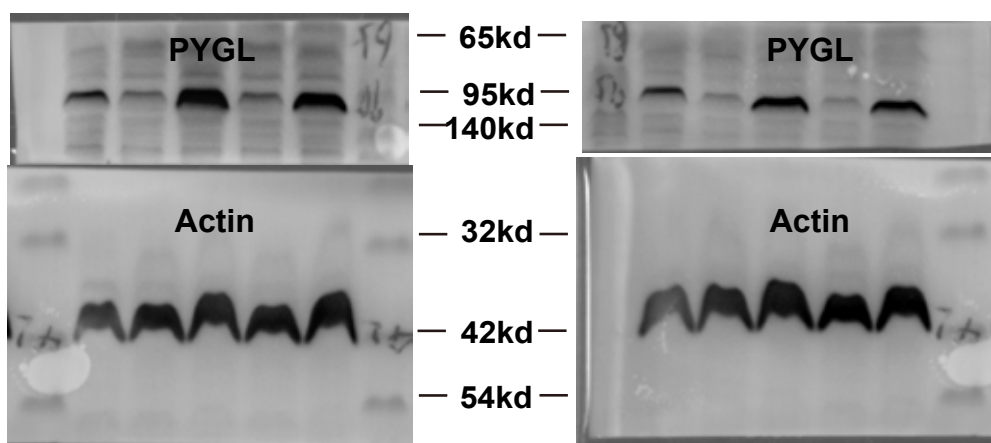

**Fig S6A**

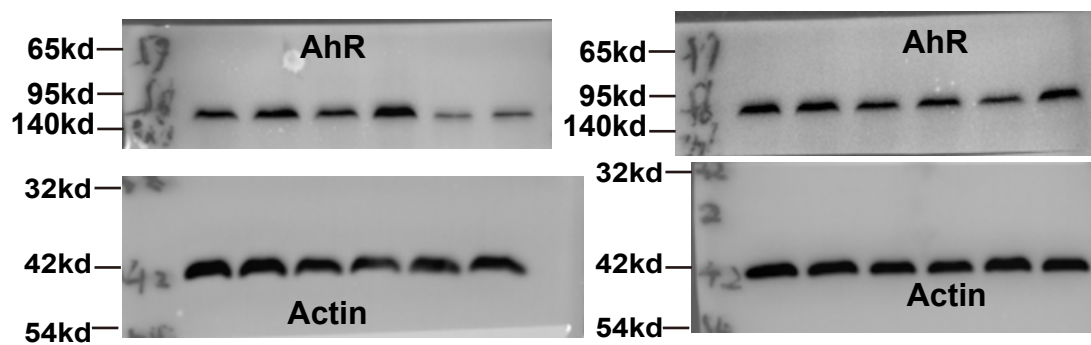

**Fig S8A**

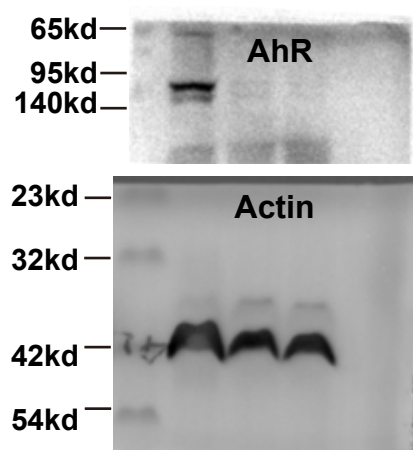

Supplement: Supplementary file 2 — the original western blot [file 41419_2026_8736_MOESM2_ESM.pdf]
